# Hidden biodiversity in microarthropods (Acari, Oribatida, Eremaeoidea, Caleremaeus)

**DOI:** 10.1038/s41598-021-02602-7

**Published:** 2021-11-30

**Authors:** Andrea Lienhard, Günther Krisper

**Affiliations:** Institute of Biology, University of Graz, Universitätsplatz 2, 8010 Graz, Austria

**Keywords:** Phylogenetics, Taxonomy, Evolution, Zoology

## Abstract

A challenge for taxonomists all over the world and across all taxonomic groups is recognizing and delimiting species, and cryptic species are even more challenging. However, an accurate identification is fundamental for all biological studies from ecology to conversation biology. We used a multidisciplinary approach including genetics as well as morphological and ecological data to assess if an easily recognizable, widely distributed and euryoecious mite taxon represents one and the same species. According to phylogenetic (based on mitochondrial and nuclear genes) and species delimitation analyses, five distinct putative species were detected and supported by high genetic distances. These genetic lineages correlate well with ecological data, and each species could be associated to its own (micro)habitat. Subsequently, slight morphological differences were found and provide additional evidence that five different species occur in Central and Southern Europe. The minuteness and the characteristic habitus of *Caleremaeus monilipes* tempted to neglect potential higher species diversity. This problem might concern several other “well-known” euryoecious microarthropods. Five new species of the genus *Caleremaeus* are described, namely *Caleremaeus mentobellus* sp. nov., *C. lignophilus* sp. nov., *C. alpinus* sp. nov., *C. elevatus* sp. nov., and *C. hispanicus* sp. nov. Additionally, a morphological evaluation of *C. monilipes* is presented.

## Introduction

The correct identification of species is the basis for studies concerning genetics, biodiversity, biogeography, or ecology, even, the definition of a species still remains a controversial topic in evolutionary biology^1^. The increased availability of genetic methods in the past two decades had led to another additional discussion: those of cryptic species. Cryptic species are defined as two (or more) distinct species that are erroneously classified as a single one^2^. Delimitation and identification of small arthropod species is mostly based on the discrimination of external morphology, because data relying on ecology, behaviour or internal characteristics for example, is difficult to gain. Speciation, however, is not always accompanied by morphological differences^2^, and consequently, reliable species delimitation is not feasible based on morphological criteria alone. Moreover, many small arthropods are difficult to identify morphologically due to the absence of distinct morphological traits. For the detection of cryptic species, interdisciplinary approaches are essential^3–5^. Schlick-Steiner et al*.*^6^ demonstrated the importance of utilizing integrative taxonomy with independent data (genetical, morphological, ecological, physiological etc.) for separating species and stated that any uni-methodical approach is supposed to have an extremely high failure rate in species delimitation.

More and more cryptic species in different taxonomic groups were recognized and this also applies to small-sized arthropods^7–11^. Mites (Acari) are the most abundant and species‐rich group of arthropods in soil and are characterized as a hyper-diverse invertebrate group, even though, the assessments of their diversity have been impeded by their small size and cryptic morphology. Mites remain poorly known, but in recent years more and more studies revealed that mainly morphology-based taxonomic knowledge has substantially underestimated mite diversity by neglecting cryptic taxa. After a literature review Skoracka et al*.*^3^ ascertained that cryptic species have been found in 17% of mite superfamilies, and Blattner et al*.*^12^ showed, that a high proportion of morphologically identified species in water mites (Hydrachnidia) appeared to be more diversified than had been assumed. Young et al*.*^13^ detected BINs (barcode index numbers) resembling 2.4 × the number of mite species recorded from Canada by means of a barcoding investigation. Young et al*.*^14^ highlighted that within hyperdiverse arthropod groups such as mites, 34% of morphospecies showed high intraspecific genetic distances and likely reflect cryptic diversity. Consequently, in many conducted studies on Acari, cryptic diversity was uncovered by high genetic distances, and subsequently morphological and/or ecological differences were found^15–20^.

Acari colonise nearly all habitats on earth and show all varieties of lifestyles. Actually, this species rich taxon comprises more than 50,000 described species^21^, but the estimated biodiversity may even exceed one million species^22^. Within the Acari the suborder Oribatida contains numerous euryoecious species with apparently worldwide occurrence^23^. The family *Caleremaeidae* belongs to the higher oribatid mites (Brachypylina). The genus *Caleremaeus* was recently redescribed^24^ and comprises four valid species: *Caleremaeus monilipes* (Michael, 1882)^25^ from the Palaearctic; *C. retractus* (Banks, 1947)^26^, *C. arboricolus* Norton & Behan-Pelletier, 2020^24^, and *C. nasutus* Norton & Behan-Pelletier, 2020^24^ from North America. Additionally, one fossil member from Baltic amber is known (Priabonian, 38–33.9 Ma in age), namely *C. gleso* Sellnick 1931^27^. Further, *Caleremaeus divisus* Mihelčič, 1952^28^ from Austria is listed as “species inquirenda”^23^ due to an insufficient description.

Despite its relative minuteness (~ 400 µm), *C. monilipes,* recently redescribed by Seniczak & Seniczak^29^, can easily be determined due to its unique habitus. This species is characterized by two pairs of prodorsal costulae, a drop-like median elevation of the notogaster and legs with globular shaped segments (monile = necklace; pes = foot). Therefore, *C. monilipes* is considered to be a “well-known species” and can be frequently recorded in the Palaearctic region. The distribution ranges from England, to northern—(Scandinavia, Sweden, Finland, Norway), eastern—(Russia, Caucasia), and southern—(Greece, Iberia, Turkey, Macaronesia) parts of Europe^23,30–33^. Moreover, this species has been found in North Africa^34^.

*Caleremaeus monilipes* represents a euryoecious species and was recorded in Europe from alluvial forests, alpine meadows, spruce forests, deciduous forests, dry grassland, scree slopes etc.^30,35–37^. Moreover, this species not only occurs in different habitats, but also in diverse substrates such as soil, litter, mosses, lichens, decaying wood and algae. Furthermore, *C. monilipes* shows a great vertical distribution from colline to alpine regions (> 2600 m asl in Austria^38^).

A publication by Lienhard et al*.*^39^ indicated a high uncorrected genetic distance in mitochondrial (> 20%) and nuclear (> 3%) gene fragments between two individuals of *C. monilipes*. Due to this fact, we attempt to verify if this easily recognizable, common, widespread and euryoecious oribatid mite belongs to one and the same species or represents a cryptic species complex. By means of an integrative approach based on mitochondrial and nuclear markers, species delimitation methods, morphological analyses and ecological investigations, the diversity among *C. monilipes* specimens from different habitats as well as substrates in Central and Southern Europe is examined.

## Results

### Molecular phylogenetic analyses

**Figure 1:**
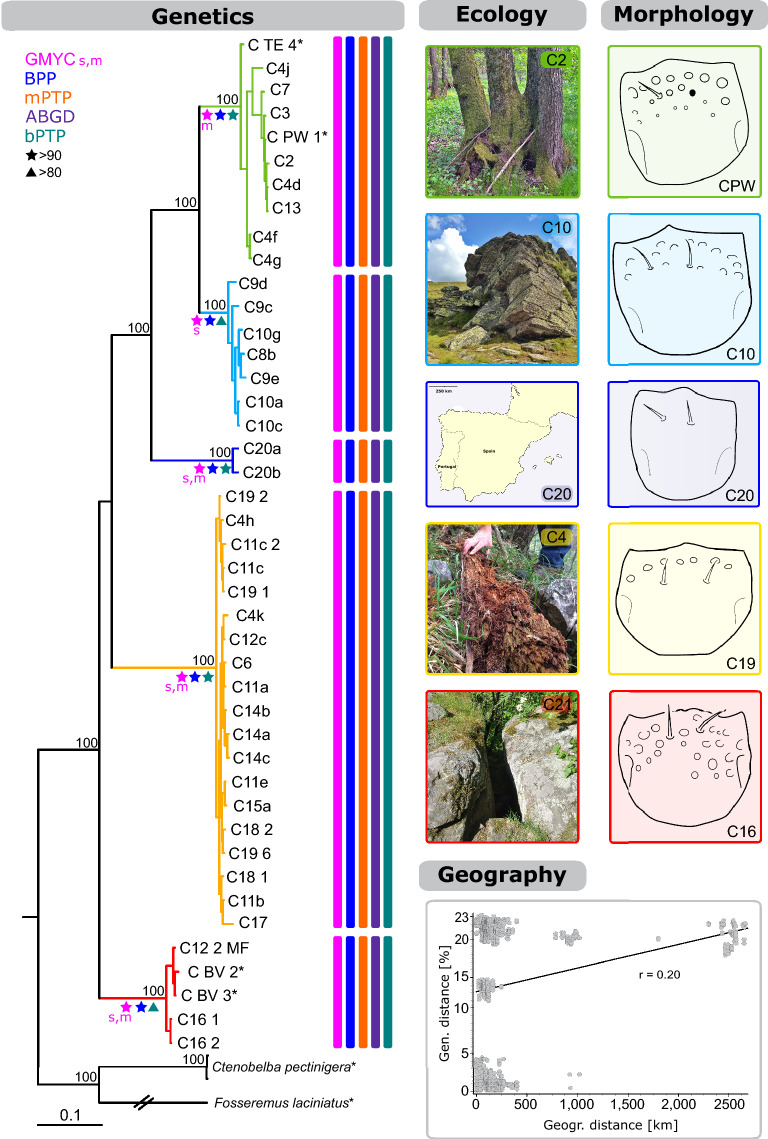
Bayesian inference tree based on the concatenated dataset (COI and EF-1α, N = 46) with the summary of species delimitation analyses, ecological characteristics, geographic and genetic correlation, and morphological differences for *Caleremaeus* specimens. Posterior probabilities (> 80) for main splits are indicated by numbers near the nodes. An asterisk marks sequences obtained from GenBank (*Lienhard et al*.*^39^). Vertical bars at terminal branches specify delimited species obtained from five different approaches. Yule support values of the GMYC analysis (m = multiple, s = single), Bayesian support values (bPTP), and posterior probabilities (BPP) > 80 are indicated by colored stars and triangles. High values indicate all descendants from this node are more likely to be from one species. Ecology: Pictures display examples of habitats specimens comprising different clades were found in. For the Spain clade no picture was available, substitutionally an image showing the sampling location is given. Morphology: Drawings show ventral mouthparts (menta) with characteristic arrays of foveae. Geography: Result of Mantel test of isolation by distance for *Caleremaeus* specimens based on the COI dataset showing the correlation of genetic (given in %, N = 71) and geographical distances (km). Correlation of genetic and geographical distances: r = 0.20. The map of the Iberian Peninsula in this figure was created with CorelDraw Graphic suite X7 (https://www.coreldraw.com).

All constructed COI gene trees and trees based on the concatenated dataset revealed five distinct *Caleremaeus* lineages, provided with high statistical support—hereafter named euryoecious, alpine, Spain, deadwood, and moss (Fig. 1). The consensus tree of the COI gene fragment inferred by four tree building methods (NJ, BI, ML, MP) can be found in Supplementary Information Online (Fig. S1). Alpine, Spain and euryoecious lineages comprise a monophyletic group. Only the position of moss and deadwood clades varied in the constructed COI trees. In BI and ML analyses the moss/deadwood clade appears as a monophyletic group, contrastingly, in MP and NJ topologies, the moss/deadwood lineage is paraphyletic. Additionally, for the COI and the EF-1α gene trees, the BI topology is shown due to the highest statistical support in the Supplementary Information online (Figs. S2, S3; the result of the SH test revealed the BI topology as most accurate for the COI dataset Table S1). The EF-1α gene alone was not able to separate alpine from euryoecious specimens, and the Spain lineage is also embedded in the alpine/euryoecious clade (Supplementary Information Online Fig. S3).

**Figure 2:**
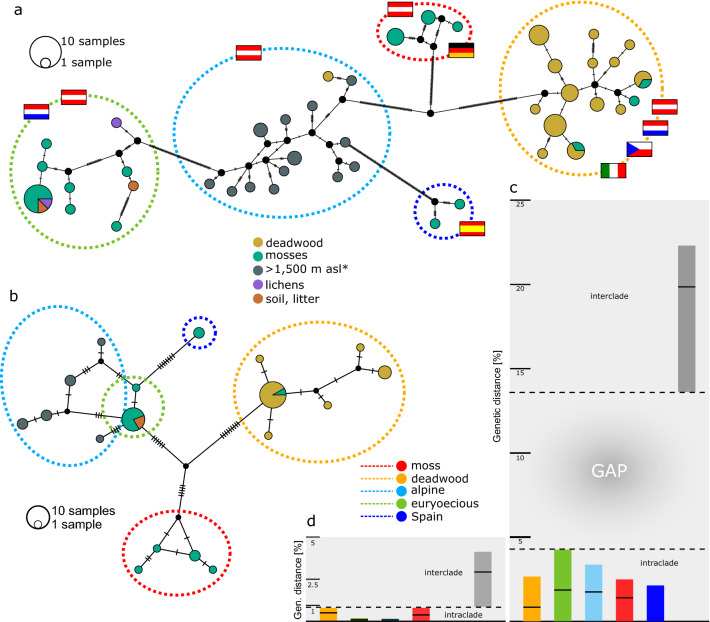
Haplotype networks and genetic distances for mitochondrial (**a**, **c**) and nuclear (**b**, **d**) genes. TCS Network of (**a**) mtDNA dataset (N = 70) and (**b**) nuDNA dataset (N = 45). Colors of circles show ecological traits and colored, dashed borders correspond to species clades detected in Fig. 1. Flags show country of origin specimens of clades were sampled in (Austria, Germany, Italy, Czech Republic, Spain, and Croatia). Each circle corresponds to one haplotype and its size is proportional to its frequency, the number of mutations between haplotypes are indicated as hatch marks. Small black circles represent intermediate haplotypes not present in the dataset. Minimum, maximum, and mean intercladal (gray) and intracladal (colors refer to clades defined in Fig. 1), uncorrected genetic distance (*p*-distance) in percent given for the (**c**) mtDNA dataset and (**d**) nuDNA dataset. Further information on mean uncorrected distances are given in Supplementary Information online (Table S3). *found in various substrates.

The haplotype network analyses were constructed based on the definition of five ecological traits in the following order (Fig. 2a, b); (i) all individuals sampled in deadwood (some were sampled > 1500 m asl), (ii) specimens found > 1500 m asl (from various substrates), and specimens sampled in (iii) mosses, (iv) lichens, or (v) litter and soil (< 1500 m asl). These ecological groups correlate well with the genetic lineages. Only one individual occurred in deadwood and was found > 1788 m asl, and three individuals were found in mosses and grouped with the deadwood clade. Remarkably, haplotypes are shared among individuals from different countries, e.g., Croatia & Austria, Czech Republic & Austria, or Italy & Austria. This fact is also underlined by the Mantel test, which indicated a weak correlation between the geographical and the genetic distance (no biogeographic pattern, Fig. 1). A total of 44 *Caleremaeus* COI haplotypes were identified. As shown in Fig. 2c, a clear gap between intracladal and intercladal *p*-distances was revealed for the COI gene fragment. The highest intracladal value (within the euryoecious clade originating from the same sampling locality) reached 4.2% (average distance: 1.6%) and the lowest intercladal value between an alpine and a euryoecious specimen (TE4 and C9e) amounted to 11.9% (mean: 19.8%). A gap was also visible for the EF-1α gene fragment (Fig. 2d), although intracladal as well as intercladal distances were generally much lower. The mean intracladal distance amounted to 0.5% and the mean intercladal distance reached 2.9%. A list of uncorrected, mean distance values for both fragments can be found in Supplementary Information Online (Table S2).

### Species delimitation analyses

All species delimitation analyses recovered five putative *Caleremaeus* species (Fig. 1). BPP and bPTP analyses supported all five delimited species with posterior probabilities > 90 and Bayesian support values > 80, respectively. For GMYC analyses the single- as well as the multiple-threshold setting revealed five putative species, although for the single threshold the euryoecious clade and for the multiple method the alpine lineage showed Yule support values < 80. In summary, delimitations were congruent across distance- and phylogeny- based approaches and confirmed five putative *Caleremaeus* species with high statistical support.

### Ecology

The five genetic lineages correlate well with ecological data and could be assigned to certain habitats and/or microhabitats (Fig. 2a). Deadwood specimens were sampled from 300 to 1750 m and appear solely in deadwood. For their occurrence, only the availability of deadwood was crucial—different kinds of wood (*Alnus*, *Picea*, *Pinus*, *Fagus, Abies* etc.), the stage of decay or the surrounding habitat (alpine meadows, marshes, spruce forests) played a negligible role. *C. monilipes* was found by Michael also in decaying wood. All specimens within the alpine clade were exclusively found above 1500 m asl in different substrates (mosses, soil, litter, lichens), but not in deadwood. Specimens within the euryoecious clade were sampled from 424 to 950 m asl and were found in diverse substrates such as soil, litter, mosses, lichens, as well as in different habitats (dry grassland, alluvial forests, mixed deciduous forests). Individuals within the moss clade were sampled from 130 to 700 m and were only found in mosses and lichens in elevated positions (on trees, rocks, boulders, roofs), but never on the ground. Only three exceptions could be made: individual C9f. was the only *Caleremaeus* individual found in a whole deadwood sample and clustered within the alpine clade. Further, individual C14b (from Dreistetten) was found in mosses on a rock and grouped with deadwood individuals (in a distance of about 20 cm individuals comprising the deadwood clade were sampled). Additionally, two individuals from location C26b (Croatia) were found in mosses on rocks and boulders, although in close vicinity (about two cm) of a decaying log. These mentioned individuals could be unambiguously determined as alpine and deadwood specimens, respectively, through morphological examination after the DNA extraction. Pictures of typical habitats are shown in Fig. 1.

### Morphological analyses

The original description of *C. monilipes* given by Michael (1882) does not contain enough detailed information on morphological characters to get sure which of the specimens we studied could be *C. monilipes*. Therefore, the Museum of Natural History, London provided us two slides of Michael’s collection (labelled as: *Notaspis monilipes* Bred., 1930.8.25.707; *Notaspis monilipes* Parts (exo), 1930.8.25.709). None of our genetically studied specimens, which we could assign to five different lineages, are morphologically identical with *C. monilipes* specimens preserved in Michael’s slides. Therefore, based on the morphological characters, the specimens in Michael’s slides represent a distinct species—the “real” *C. monilipes*.” As the morphological analyses confirm the five lineages determined by genetic investigations we established the following five new species (for details of type series see “Material and methods”).

- *Caleremaeus alpinus* sp. nov. (= alpine lineage).
- *Caleremaeus elevatus* sp. nov. (= moss lineage).
- *Caleremaeus hispanicus* sp. nov. (= Spain lineage).
- *Caleremaeus lignophilus* sp. nov. (= deadwood lineage).
- *Caleremaeus mentobellus* sp. nov. (= euryoecious lineage).

**Figure 3:**
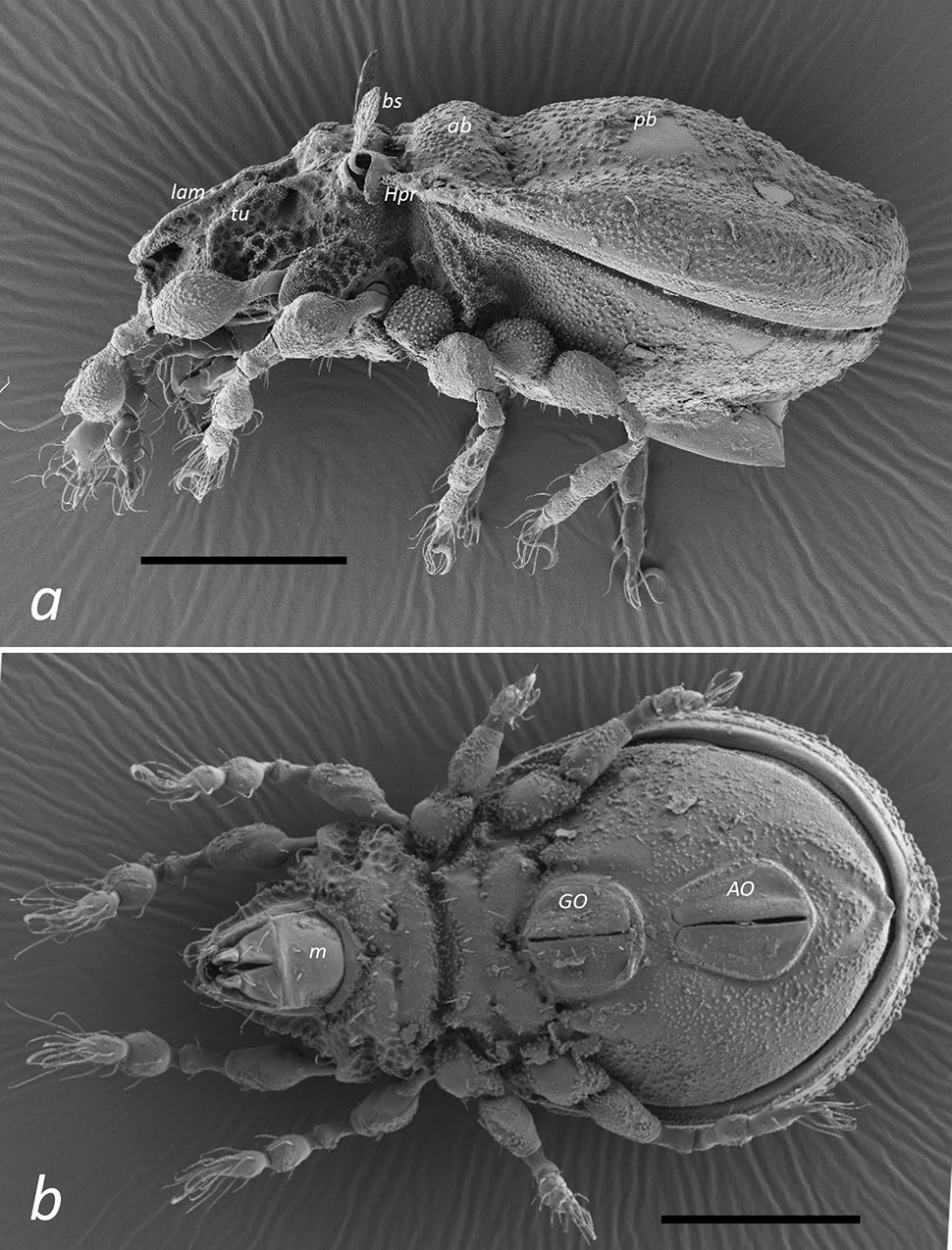
SEM micrographs of (**a**) *Caleremaeus mentobellus* (lateral) and (**b**) *C. hispanicus* (ventral). lam = lamella, tu = tutorium, bs = bothridial seta, Hpr = humeral process, ab = transverse anterior bulge of notogaster, pb = longitudinal posterior bulge of notogaster, m = mentum, GO = genital orifice, AO = anal orifice; scale bars = 100 µm.

The typical appearance of *Caleremaeus* species described in our study is shown in Fig. 3, using the example of *C. mentobellus* and *C. hispanicus*.

**Figure 4:**
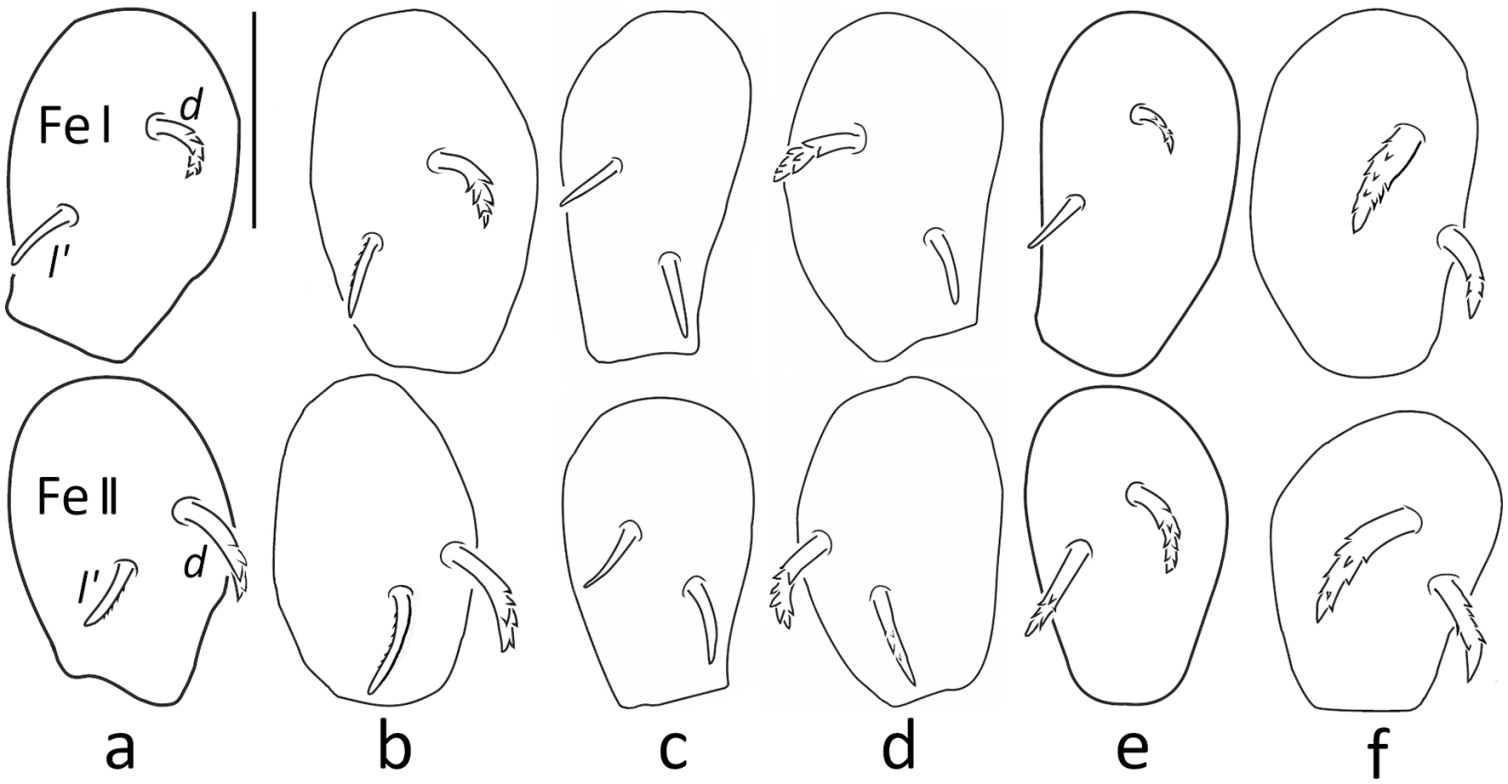
Comparison of dorsal setation of femora I and II (proximal parts of femora not drawn). (**a**) *Caleremaeus mentobellus*, (**b**) *C. lignophilus*, (**c**) *C. elevatus*, (**d**) *C. alpinus*, (**e**) *C. hispanicus* and (**f**) *C. monilipes*; scale bar = 25 µm.

**Figure 5:**
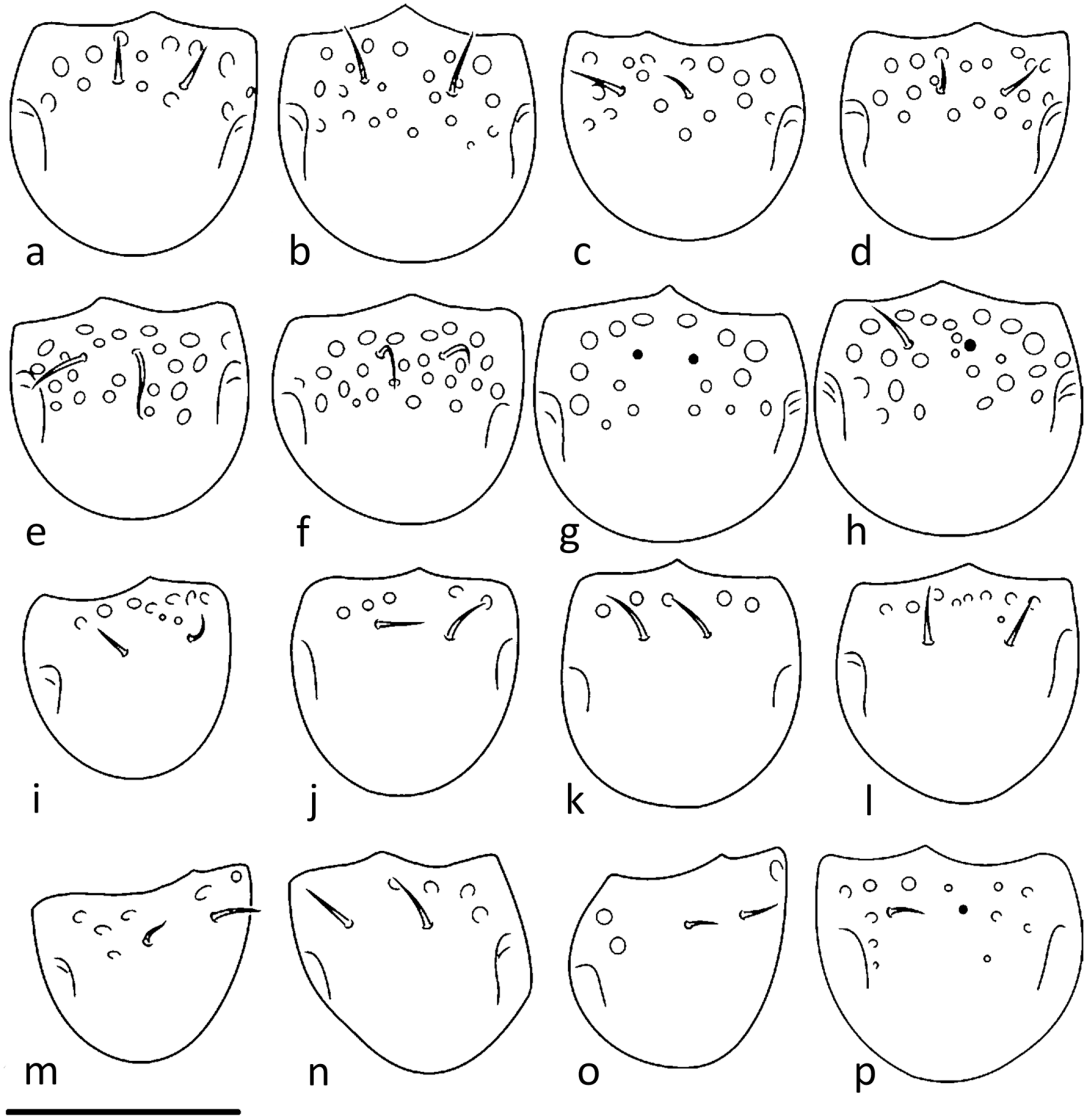
Inter- and intraspecific variation of foveae on mentum of different *Caleremaeus* species; some menta in ventrolateral view. (**a**–**d**) *Caleremaeus mentobellus*: (**a**) Peggau, (**b**) Klafferbach valley, (**c**) Deutschlandsberg, (**d**) Lučice (HR); (**e**–**h**) *C. elevatus*: (**e**,**f**) Murau, (**g**) Bad Ischl, (**h**) Burg Eltz (D); (**i**–**l**) *C. lignophilus*: (**i**) Heimschuh, (**j**) Tamischbachgraben, (**k**) Scheiblalm, (**l**) Pontebba (I); (**m**–**p**) *C. alpinus*: (**m**–**o**) Handalpe, (**p**) Saualpe; scale bar = 50 µm.

**Figure 6:**
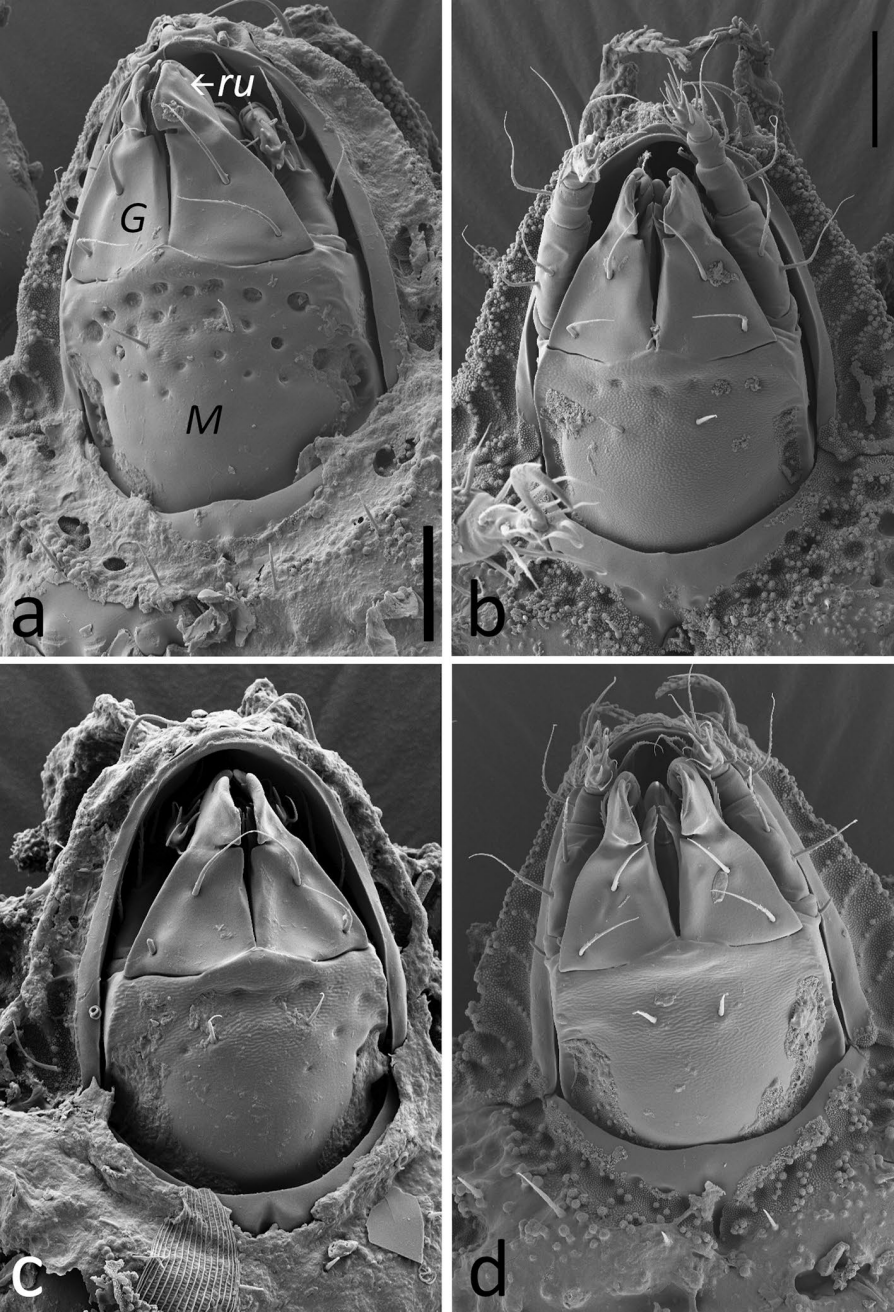
SEM micrographs of the distribution of foveae on mentum. (**a**) *Caleremaeus mentobellus*, (**b**) *C. lignophilus*, (**c**) *C. alpinus*, (**d**) *C. hispanicus*; M = mentum, G = gena, ru = rutellum. Scale bar = 20 µm.

In the following, important diagnostic characters for species discrimination are compared in Table 1 and Figs. 4, 5 and 6 including data of *C. monilipes.* More detailed descriptions with further species-specific characters (for instance the structure of the cerotegument) are given in the Supplementary Information online (part 2). Whereas the body size is not suitable for species discrimination, the features distinguishing the species mainly concern inconspicuous differences of morphological structures and their combination. Especially cuticle surface structures of the mentum and the shape of femoral setae *d* and *l*′ of legs I and II are important. In their redescription of *C. monilipes* Seniczak & Seniczak^29^ mentioned (p. 1996): “most leg setae relatively short and smooth, except barbed seta *d* on all femora” (similar to *C. mentobellus*). This fact does not correspond to the situation found in the slides of *C. monilipes* (Michaels’ collection), where the setae *d* and *l’* on the dorsal side of femora I and II are very stout and barbed. Another difference concerns the bothridial enantiophyses: one in Seniczak & Seniczak^29^ but two in Michael’s slides; furthermore, the tutorium on the lateral side of the propodosoma is weakly developed, but in Michael’s specimens and the herein described species it is a very pronounced structure. The mentum depicted in the latter mentioned paper shows no foveae which would correspond to Michael’s *C. monilipes.* The character ‘rows of foveae on mentum’ may be difficult to assign to a certain species because of intraspecific variation in the distribution of the foveae (Fig. 5). Even though the menta of *C. mentobellus* & *C. elevatus* (several rows of clearly visible foveae)*, C. lignophilus* & *C. alpinus* (one row of foveae)*,* and *C. hispanicus* & *C. monilipes* (no foveae) are similar to each other, a clear tendency of the arrangement is recognizable (Fig. 1)*.* The menta of four species, showing the structure of the cuticle surface, are additionally depicted as SEM-micrographs in Fig. 6. Each species has its own combination of characters, discrepancies in the leg setation among *C. monilipes* and all other species are shown in Table 2.

**Table 1:** Comparison of diagnostic characters of *Caleremaeus* species.

Character		*Caleremaeus*					
		*monilipes*	*mentobellus*	*lignophilus*	*elevatus*	*alpinus*	*hispanicus*
Body	Length (µm)	363	333–386	307–367	322–376	353–417	362–379
	Width (µm)	190	170–215	156–194	163–220	177–222	192–204
Mentum	Foveae rows	0	3	1	3 + f	1 + f	0
	Cuticle	⊙	◊	⊙	⊙	◊	◈
Femur I	Seta *d*	✶	♦	♦	⊚	♦	■
	Seta *l'*	✶	⊚	■	⊚	⊚	⊚
Femur II	Seta *d*	✶	♦	♦	⊚	♦	♦
	Seta *l'*	✶	■	■	⊚	■	♦
Bothridial enantiophyses		1	2	2	2	2	2
Genital setae		6 + 6	6 + 6	6 + 6	5 + 5	6 + 6	6 + 6

f = few foveae laterally; ⊙ = finely granulate, ◈ = finely rugose, ◊ = finely rugose in anterior half of mentum, ✶= very stout and spinose, ♦ = stout and spinose, ⊚ = spiniform and smooth, ■ = spiniform with tiny barbs.

**Table 2:** Comparison of leg setation of all recent *Caleremaeus* species.

Species	Chaetotaxy trochanter to tarsus			
	Leg I	Leg II	Leg III	Leg IV
*Caleremaeus monilipes* Michael	1-4-3-4-19	1-4-2-4-15	2-3-1-3-13	1-1-2-3-11
*C. mentobellus* sp. nov	1-4-3-4-20	1-4-2-4-16	2-3-1-3-14	1-2-2-3-11
*C. alpinus* sp. nov	1-4-3-4-20	1-4-2-4-16	2-3-1-3-14	1-2-2-3-11
*C. lignophilus* sp. nov	1-4-3-4-20	1-4-2-4-16	2-3-1-3-14	1-2-2-3-11
*C. elevatus* sp. nov	1-4-3-4-20	1-4-2-4-16	2-3-1-3-14	1-2-2-3-11
*C. hispanicus* sp. nov	1-4-3-4-20	1-4-2-4-16	2-3-1-3-14	1-2-2-3-11
*C. monilipes* sensu Seniczak	1-4-3-4-20	1-4-3-4-15	2-3-1-3-14*	1-2-2-3-11*
*C. retractus*, *arboricolus*, *nasutus*	1-4-3-4-20	1-4-2-4-16	2-3-1-3-14	1-2-2-3-11

*Corrected in Norton & Behan-Pelletier^24^.

## Discussion

The results of this integrative approach revealed congruent results based on genetic data, species delimitation analyses, morphological investigations, and ecological traits (Fig. 1). Several authors claimed that delimitations based on morphologic or genetic data alone are likely inadequate and species delimitation should be conducted with consideration of the ecology, behaviour, the life history, and geographical distribution, respectively^2–4,40,41^. Moreover, Carstens et al*.*^40^ asserted that it is important to analyse data with a wide variety of methods for delimitation (researcher present results from 2.25 methods on average), because any existing method is forced to make a series of simplifying assumptions, therefore there is no ideal method. In our case, all species delimitation analyses provide consistent results, confirming that data is solid, and the status is beyond doubt: the lineages are clearly five separate *Caleremaeus* species. The agreement across other independent data sources further strengthens the confidence in these results and the subsequent species delimitations. The mean intracladal *p*-distance of the COI gene amounted to 1.6% versus 19.8% intercladal distance (Fig. 2). Hebert et al*.*^42^ proposed that 10× the mean ratio of intra- to interspecific variation in COI sequences can sanction provisional species distinction. Therefore, our study supports the efficacy of the COI gene in oribatid mites in the identification of species boundaries. It was shown that a divergence rate of 2.15% is applicable for time estimations for the COI gene in Oribatida^15,43^. Given this rate all species would have separated about 6–10 million years ago. Although the nuclear marker mutates at a much slower rate than the mitochondrial DNA, a gap between mean intra- and interspecific distances was present. Based on phylogenetic trees, however, the EF-1α has not yet accumulated the differences needed to delimitate all five species.

Most mite species known today have been described based entirely on external morphology, genetic methods to verify and complement these descriptions are still relatively scarce. These morphology-based identifications can be prone to difficulties: (i) the lack of diagnostic traits, or (ii) characters displaying phenotypic plasticity (intraspecific variation) which do not resemble species borders^8,41^. Ad (i) Speciation is not necessarily accompanied by obvious morphological differentiation even though the entities might be genetically isolated^2^. Within Oribatida the morphology has been proven over millions of years and it is expected that cryptic species are especially common in Acari because of the phenomenon of morphological stasis (= retention of same ancestral character state over an extended period of time)^3^. Most mite species are virtually blind and mate recognition is frequently based on cues other than morphological traits^2,3^. Thus, many oribatid mite species are indistinguishable on the basis of morphological criteria alone and are most likely to be uncovered by genetic investigations. The highly conserved morphology observed among *Caleremaeus* species suggests the occurrence of morphological stasis. *Caleremaeus gleso* from Eocene Baltic amber underlines this assumption; it displays only little morphological variation over millions of years and can be unambiguously identified as a member of the genus *Caleremaeus* based on the drawing in the description of Sellnick^27^. Ad (ii) Our integrative analysis enabled an evaluation of the phylogenetic value of available taxonomic characteristics and identified those with the most power to discriminate among species of the genus *Caleremaeus*. Only a detailed morphological study, in the light of the outcome of molecular analyses, allowed us to detect taxonomic traits that characterize these species (Table 1). In *Caleremaeus,* intraspecific variation concerns the foveae on mentum (Fig. 5), but the general arrangement or the absence of these foveae represents a valuable character to differentiate the species described in this study (Fig. 1). The search for these subtle diagnostic morphological characters requires an expert morphologist with much experience and is very time consuming, time, young researchers often do not have, although, molecular evidence allows to avoid false inference related to these “morphology-based difficulties”.

Another dilemma concerning “cryptic species” is the fact that descriptions are often recommended by authors, but not provided. Only 25% of studies reviewed by Carstens et al*.*^40^ include a species description. Struck et al*.*^4^ also surveyed over 600 publications on cryptic species and barely 19% provided formal descriptions, and thus are valid. Even worse, 47% of all studies claiming cryptic species presented no phenotypical data^4^. Estimates of species diversity are crucial for understanding evolutionary processes, ecosystem functioning, and for developing effective conservation strategies^44^. Formal species descriptions are often “only” published in taxon focused journals rather than in those of general interest. These two journal types are not viewed as equivalent in the scientific community, although, formal species descriptions are undisputable the basis for subsequent investigations.

Tiny widespread generalist species with a characteristic habitus are prone to hide cryptic diversity. Fontaneto et al*.*^45^ presented three reasons influencing diversifications of microscopic animals. For *Caleremaeus* species only one assumption is appropriate (there might be more available niches at smaller scales due to greater spatial and temporal heterogeneity), because species have no resting stages and reproduce sexually (not parthenogenetically)^24^. Ecological parameters are difficult to evaluate, especially for tiny organisms like oribatid mites. But these parameters are important, because environmental heterogeneity on smaller scales results in more available niches for minute animals such as mites and contribute to the occurrence of more species per habitat compared to larger organisms^3^. Skoracka et al.^3^ highlighted the important role of ecology in driving speciation in mites. In fact, speciation through adaptations to different ecological niches concerning “cryptic species” was demonstrated in mites by several studies. Laumann et al*.*^46^ found two coexisting *Tectocepheus* species inhabiting different layers in soil or Pfingstl et al*.*^18^ discovered two “cryptic” species within *Carinozetes* occupying different ecological niches (mangrove roots and rocky shores). Furthermore, Martin et al*.*^47^ explored that populations of water mites inhabiting lakes and streams are two different species. Zhang et al*.*^48^ revealed cryptic diversity underlined by different habitat types in small flightless arthropods (Collembola) and in a study conducted with microscopic animals (Rotifera), species believed as generalist exhibit the highest cryptic diversity^45^. Species considered to be generalist may present complexes of cryptic taxa, each of them adapted to narrower niches^45^. Thus, the question raises of whether “cryptic species” tend to be ecological generalist or specialist^4^. According to our study a strong association of each *Caleremaeus* species to a specific microhabitat, and a clear genetic differentiation between species of neighbouring microhabitats, but not between distant microhabitats of the same type, indicate a high degree of habitat specialization. But what does euryoecious mean (“having a wide range of habitats”) in the context of *Caleremaeus*? *Caleremaeus alpinus* is restricted to subalpine and alpine habitats but can be found in various substrates and thus, can be considered as euryoecious within the habitat. *Caleremaeus lignophilus* is restricted to decaying wood (stenoecious concerning the substrate in general) but without limitation to the type of wood—and in this sense—it is euryoecious. For *C. lignophilus* and *C. monilipes,* dwelling in decaying wood, special nutritional resources may be essential factors for their distribution. Newly discovered cryptic species complexes also claimed that there are more dietary specialists than was previously thought^2^. Nevertheless, we analysed remnants in the digestive system of several *Caleremaeus* specimens; mainly fungal hyphae which could not be determined more precisely were detected in all species. “*C. monilipes*” is regarded as a macrophytophage (with the ability to digest dead or decaying tissues of higher plants, including leaves and wood^37,49^. Indeed, so far *Caleremaeus* was mostly found in decaying wood^50–52^ and high abundances of *Caleremaeus* specimens were—also in our study—only found in deadwood samples. The fact that *Caleremaeus lignophilus* and *C. mentobellus* show a syntopic occurrence (they occur in the same habitat at the same time and are closely related species) is well demonstrated by the fact that in sampled soil and litter from a *Sesleria albicans* lawn (Weizklamm) with a branch of deadwood laying directly on it, solely *C. mentobellus* and in the deadwood only specimens comprising *C. lignophilus* were found. Noteworthy is further, that individuals from Croatia (Lučice) and Austria share the same haplotype, whereas individuals from Croatia found in the same mixed sample (deadwood and mosses) are genetically clearly separated. Moreover, specimens of *C. elevatus* and *C. lignophilus* live in immediate vicinity (only a few meters apart) in the same habitat/locality (e.g., in Bad Vöslau), but were found in different substrates. The occurrence in the same locality supports the species status and indicates no or limited gene flow—species cannot or do not exchange genetic material anymore. These species apparently ceased exchanging genes, and their occurrence in syntopy underline the fact that they represent biological species. The question, which important ecological factors and adaptations are responsible for the distribution of *Caleremaeus* species, remains. *Caleremaeus elevatus* might be tolerant to extreme droughts occurring in mosses and lichens on sun-exposed rocks and roofs. In contrast, *C. alpinus* could be adapted to low temperatures and to a short active period.

As classical morphological taxonomy of microscopic organisms is often not able to resolve their actual diversity, cosmopolitan distribution is often a result of misidentifications^45^. Cosmopolitanism is considered as an exception in microorganisms (as it is in macroorganisms)^53^. Fontaneto et al*.*^45^ predicted that cryptic species in microscopic animals should not be geographically separated entities but comprise niche specialists either occupying different habitat types or co-occurring in micro-niches in the same habitat. The fact that geographical isolation has a small impact showing a weak correlation to genetic distance (Fig. 1) is not only shown in our study, but also Schaeffer et al*.*^20^ found no geographic pattern for oribatid mites. This fact is further surprising because most Oribatida have limited active dispersal capabilities because of their minute size and their winglessness. The question arises how new microhabitats can be colonised so targeted by these tiny creatures. Understanding different dispersal processes are key in understanding how communities are formed and maintained^54^. Firstly, the distinction between active and passive dispersal is important. Rates of active, cursorial transport are low for most species of oribatid mites^54^, thus, only passive transport over long distances seems to be possible by following modes: hydrochory, anemochory, and zoochory (especially bird-mediated transport). Mites in littoral habitats may favor hydrochorous dispersal, leading to an increased genetic exchange and a wide distribution for instance in the Caribbean by means of the Gulf Stream^19^. Wind dispersal on the other hand is an important dispersal mode for arboreal oribatid mites^55^ and individuals might be carried considerable distances^54^, but wind as well as water dispersal for *Caleremaeus* species is not likely. *Caleremaeus* species are not known as arboreal, although, two specimens were found in litterfall collected from canopy in 30 m height^54^. During our study, several bark samples were collected, but only two specimens were found in the bark of *Picea abies*. Nevertheless, oribatid mites are typically not known to participate actively in phoresy, but accidental phoresy may occur as there is evidence for transport on birds^54^ and *Caleremaeus* specimens were occasionally found in plumage and nests of different bird species^56–58^.

Whereas the species described in this study show subtle morphological differences, the North American *Caleremaeus* species can easily be distinguished from each other^24^. Although, Norton & Behan-Pelletier^24^ presumed that *C. retractus* may represent a species complex as demonstrated here for the European *Caleremaeus*. Their assumption is based on different specimens showing a smooth mentum or one with scattered foveae, which is, in the light of our results, a strong indication that *C. retractus* represents more than one species. Similarly, *C. monilipes* from Michael’s collection showed morphological differences (the numbers of leg setation, the form of femoral setae, the shape of seta *tc*′ of tarsus III) compared to *C. monilipes* specimens from Norway described by Seniczak & Seniczak^29^. The combination of these characters and divergent numbers in the leg setation (Table 2) let us assume that the Norwegian specimens represent another new *Caleremaeus* species. Hence, we assume that a more thorough sampling all over the Palearctic range of distribution will probably uncover numerous additional lineages within *Caleremaeus*.

Threatened species within cryptic complex require new conservation statuses. As many mite species are valuable bioindicators^59^, have impacts on human health, on agriculture or on ecological functions like decomposition, the recognition of (cryptic) species is not only important for biodiversity estimations, but also for conservation, safety, and environmental purposes. Stenotopic *C. alpinus* tolerates a narrow range of environmental changes and might be affected by climate warming, or *C. lignophilus* might play an important role in the decomposition of deadwood. Generally, Oribatida are essential decomposers, but their unique role in an ecosystem is often not yet detected.

Cryptic diversity can be overlooked especially in taxa with a typical and easy recognizable habitus. Consequently, such cases tempt not to study subtle morphological details to detect differences. Concerning our study, although vast genetical disparity and obvious results of species delimitation analyses are present, morphological differences as the foveae on menta or fine structures of leg setae can only be detected by preparing microscopic slides or SEM-micrographs. Although, several authors noted morphological disparity within *C. monilipes* or declared “*Caleremaeus* sp.”^29,30,33,35–39,60–64^, the characteristic habitus tempts to neglect potential higher species diversity. This problem might concern several other “well known” taxa as well. As Struck et al*.*^4^ (p. 155) stated, “If biologist cannot even agree on what to consider different species, then how can we reach consensus on what represents cryptic species?” Even if cryptic species are only a temporary taxonomical problem of species delineation and nothing more than an incompatibility of species “concepts” or if they are a natural phenomenon^65,66^, the species “problem” still remains the “mystery of mysteries”^67^, but scientists do their best to “throw some light on the origin of species”.

## Material and methods

### Sampling

*Caleremaeus* specimens investigated in this paper were collected at 60 different localities in Austria, Germany, Italy, Czech Republic, Croatia, and Spain between 1983 and 2016, to perform genetic and morphological investigations (Supplementary Information Online Table S3). Specimens were extracted from decaying wood, *Loiseleuria procumbens*, mosses, lichens or from soil and litter samples using Berlese-Tullgren funnels. Animals were collected in polystyrol-boxes with a base of moistened plaster of paris to get specimens alive for later preservation in absolute ethanol.

### Genetic analyses

#### Extraction, PCR and DNA sequencing

In total, 70 specimens of *Caleremaeus* spp. were genetically analysed. Extraction of genomic DNA was carried out by a genomic DNA kit (NucleoSpin Tissue XS by Macherey–Nagel, modifications see Lienhard et al*.*^68^). Therefore, total genomic DNA was extracted from single individuals preserved in absolute ethanol. Two gene fragments were sequenced for this study: the mitochondrial *cytochrome c oxidase subunit 1* gene (COI), the nuclear *elongation factor 1 alpha* gene (EF-1α). A 567 bp fragment of the COI gene was amplified using the primer pairs Mite COI 2F and Mite COI 2R^69^, and for amplifying 513 bp of the EF-1α gene, the primers 40.71F and 52.RC^70^ were used. PCR conditions for the COI gene fragment are given in Pfingstl et al*.*^16^ and those for the EF-1α gene fragment in Lienhard et al*.*^39^. DNA purification (with the enzyme cleaner ExoSAP-IT, Affymetrix; and the Sephadex G 50 resin, GE Healthcare) and sequencing steps (using the BigDye Sequence Terminator v3.1 Cycle Sequencing Kit, Applied Biosystems) were conducted after the methods published by Schaeffer et al*.*^71^. Sequencing was performed in both directions and sequences were visualized on an automated capillary sequencer (ABI PRISM 3130xl, Applied Biosystems).

#### Data analysis

Electropherograms very checked by eye and sequences were aligned using MEGA version 7^72^. Gene fragments were analysed individually and as a concatenated dataset comprising mtDNA and nucDNA (COI and EF-1α, 1080 bp). The best fitting models of molecular evolution (COI: GTR + I + G; EF-1α: GTR + G) were selected based on the AIC (Akaike Information Criterion) in the smart model selection (SMS^73^, in PhyML^74^; http://www.atgc-montpellier.fr/sms/).

For both gene fragments and for the concatenated dataset, Bayesian 50% majority rule consensus trees were generated by means of MrBAYES 3.1.2^75^ applying a MC^3^ simulation with 10–20 million generations (6 chains, 2 independent runs, 10% burn-in, Supplementary Information online Figs. S1–S3). Results were analysed in TRACER v.1.7.1^76^ to check for convergence and to ensure the stationarity of all parameters. BI gene trees of the mtDNA and the nucDNA dataset are given in Supplementary Information online (Figs. S2, S3).

For the COI dataset additional phylogenetic trees, utilizing single tree building algorithms (Neighbor joining, maximum parsimony and maximum likelihood) as well as a consensus tree (75% majority-rule) from four tree building methods (NJ, BI, ML, MP) were constructed (further information: Supplementary Information Online, part 1).

Uncorrected *p*-distances were calculated in MEGA7 and TCS Networks^77^ were constructed by means of the program PopART applying default settings^78^ http://popart.otago.ac.nz). To determine the geographic correspondence with the genetic structure a Mantel test as implemented in Alleles in Space 1.0 (AIS^79^) was performed.

All sequences obtained from this study were deposited in GenBank (www.ncbi.nlm.nih.gov/genbank; accession numbers for COI: OK545907–OK545972 and EF-1α: OK545973–OK546011; more details are given in the Table S4). Additionally, all available GenBank sequences of *Caleremaeus* specimens of the COI and EF-1α gene were integrated in the alignment as well as several outgroup taxa.

#### Species delimitation

Species delimitation was performed by applying five different methods. Both, distance- and phylogeny- based approaches were used, namely the (1) general mixed Yule coalescent model (GMYC^80^); the (2) Poisson Tree Processes model for species delimitation (PTP^81^); the (3) Automatic Barcode Gap Discovery (ABGD^82^); the (4) multi rate Poisson Tree Processes (mPTP^83^); and the (5) Bayesian Phylogenetics and Phylogeography (BPP^84,85^). Further details on these methods are given in the Supplementary Information online.

### Morphological investigations

For investigations in transmitted light in permanent slides specimens were preserved in ethanol (70%) and then embedded in Hoyer’s medium. Investigations and drawings were conducted with a differential interference contrast microscope (Olympus BH-2) equipped with a drawing attachment.

For SEM-micrographs specimens were dehydrated in ascending ethanol concentrations, dried on air, and mounted on aluminium-stubs with double sided sticky carbon tape. At last, the specimens were sputter-coated with gold. SEM-micrographs were either made at the Institute of Plant Sciences, University of Graz with a Philips XL30 ESEM or at the Research Institute for Electron Microscopy and Fine Structure Research, Graz, University of Technology, with a Zeiss Leo Gemini DSM 982.

Holotypes and paratypes of the five new species are deposited in the Senckenberg Museum of Natural History, Görlitz.

*Caleremaeus alpinus* sp. nov.; holotype (Colnr. 64289) and eight paratypes preserved in ethanol (Colnr. 64290) and three paratypes as microscopic slides (Colnrs. 64291–64293). The type material comes from mosses out of an alpine meadow (N 46.844250, E 15.019639; Austria, Styria, Handalpe, 1811 m asl. 17/07/2014).

*Caleremaeus elevatus* sp. nov.; holotype (Colnr. 64294) and one paratype as microscopic slide (Colnr. 64295), and eight paratypes in ethanol; (Colnr. 64296). The type material mounted in the slides comes from dry mosses on rocks (N 50.205190, E 7.336211; close to the Castle Eltz, Germany; 130 m asl. 21/08/2014, E. McCullough leg.); the paratypes preserved in ethanol originate from mosses of a wooden shingle roof (N 47.098889, E 13.992778; Austria, Styria, Falkendorf near Stadl/Mur, ~ 1110 m asl. July 1988, R. Schuster leg.).

*Caleremaeus hispanicus* sp. nov.; holotype (Colnr. 64297) and two paratypes in ethanol (Colnr. 64298) and three paratypes as microscopic slides (Colnrs. 64299–64301). The type material comes from mosses in a beech forest (N 42.623611, E − 7.031389; Busmayor, Barjas, Castile and León, Spain; 1300 m asl. 19/10/2014, M. Guerra leg.).

*Caleremaeus lignophilus* sp. nov.; holotype (Colnr. 64302) and ten paratypes in ethanol (Colnr. 64303). The type material comes from dead wood of a mixed deciduous forest (N 47.084324, E 15.462496; Austria, Styria, Graz, Leechwald, 400–450 m asl. 05/08/2014).

*Caleremaeus mentobellus* sp. nov.; holotype (Colnr. 64304) and five paratypes preserved in ethanol (Colnr. 64305) as well as two paratypes as microscopic slides (Colnrs. 64306–64307). The type material comes from mosses in a mixed deciduous forest (N 47.270828–47.269960, E 15.584375–15.583504; Austria, Styria, Weizklamm, 710–790 m asl. 24/04/2014).

## Supplementary Information

Supplementary Information.

## References

[1] Zachos, F. E. *Species Concepts in Biology*. Cham, Switzerland: Springer. 10.1007/978-3-319-44966-1 (2016).

[2] Bickford D (2007). Cryptic species as a window on diversity and conservation. Trends Ecol. Evol..

[3] Skoracka A, Magalhães S, Rector BG, Kuczyński L (2015). Cryptic speciation in the Acari: A function of species lifestyles or our ability to separate species?. Exp. Appl. Acarol..

[4] Struck TH (2018). Finding evolutionary processes hidden in cryptic species. Trends Ecol. Evol..

[5] Korshunova T (2019). Multilevel fine-scale diversity challenges the ‘cryptic species’ concept. Sci. Rep..

[6] Schlick-Steiner BC (2010). Integrative taxonomy: A multiscore approach to exploring biodiversity. Annu. Rev. Entomol..

[7] Pfenninger M, Schwenk K (2007). Cryptic animal species are homogeneously distributed among taxa and biogeographical regions. BMC Evol. Biol..

[8] Resch, M. C. *et al.* Where taxonomy based on subtle morphological differences is perfectly mirrored by huge genetic distances: DNA barcoding in Protura (Hexapoda). *PloS one* **9**(3), e90653 (2014). 10.1371/journal.pone.0090653 PMC3946556 24609003

[9] Sun X (2017). Delimiting species of *Protaphorura* (Collembola: Onychiuridae): integrative evidence based on morphology, DNA sequences and geography. Sci. Rep..

[10] Zhang B, Chen TW, Mateos E, Scheu S, Schaefer I (2019). DNA-based approaches uncover cryptic diversity in the European *Lepidocyrtus lanuginosus* species group (Collembola: Entomobryidae). Invertebr. Syst..

[11] Pfingstl, T., Lienhard, A., Baumann, J. & Koblmüller, S. A taxonomist‘s nightmare–Cryptic diversity in Caribbean intertidal arthropods (Arachnida, Acari, Oribatida). *Mol. Phylogenet. Evol.* **163**, 107240 (2021). 10.1016/j.ympev.2021.107240 34197900

[12] Blattner L, Gerecke R, Von Fumetti S (2019). Hidden biodiversity revealed by integrated morphology and genetic species delimitation of spring dwelling water mite species (Acari, Parasitengona: Hydrachnidia). Parasites Vectors.

[13] Young MR, Proctor HC, Dewaard JR, Hebert PD (2019). DNA barcodes expose unexpected diversity in Canadian mites. Mol. Ecol..

[14] Young MR (2019). Linking morphological and molecular taxonomy for the identification of poultry house, soil, and nest dwelling mites in the Western Palearctic. Sci. Rep..

[15] Heethoff M, Laumann M, Weigmann G, Raspotnig G (2011). Integrative taxonomy: combining morphological, molecular and chemical data for species delineation in the parthenogenetic *Trhypochthonius tectorum* complex (Acari, Oribatida, Trhypochthoniidae). Front. Zool..

[16] Navia D (2013). Cryptic diversity in *Brevipalpus* mites (Tenuipalpidae). Zool. Scr..

[17] Pepato AR, Vidigal TH, Klimov PB (2019). Evaluating the boundaries of marine biogeographic regions of the Southwestern Atlantic using halacarid mites (Halacaridae), meiobenthic organisms with a low dispersal potential. Ecol. Evol..

[18] Pfingstl, T., Lienhard, A. & Jagersbacher-Baumann, J. Hidden in the mangrove forest: The cryptic intertidal mite *Carinozetes mangrovi* sp. nov. (Acari, Oribatida, Selenoribatidae). *Exp*. *Appl*. *Acarol*. **63**, 481–495 (2014). 10.1007/s10493-014-9802-2 24687175

[19] Pfingstl T, Baumann J, Lienhard A (2019). The Caribbean enigma: The presence of unusual cryptic diversity in intertidal mites (Arachnida, Acari, Oribatida). Org. Divers. Evol..

[20] Schaeffer S, Kerschbaumer M, Koblmüller S (2019). Multiple new species: Cryptic diversity in the widespread mite species *Cymbaeremaeus cymba* (Oribatida, Cymbaeremaeidae). Mol. Phylogenet. Evol..

[21] Zhang ZQ (2011). Animal biodiversity: An introduction to higher level classification and taxonomic richness. Zootaxa.

[22] Walter, D. E. & Proctor, H. C. *Mites–ecology, evolution and behaviour: Life at a microscale*. 2nd edn. (Springer, The Netherlands, 2013).

[23] Subías, L. S. Listado sistimatico, sininimico y biogeografico de los Acaros Oribatidos (Acariformes, Oribatida) del mundo (1748–2002). *Graellsia* **60**, 3–305 (2004). (updated 2018).

[24] Norton, R. A. & Behan-Pelletier, V. Two unusual new species of *Caleremaeus* (Acari: Oribatida) from eastern North America, with redescription of *C. retractus* and reevaluation of the genus. *Acarologia* **60**(2), 398–448. 10.24349/acarologia/20204375 (2020).

[25] Michael AD (1882). Further notes on British Oribatidæ. J. R. Microsc. Soc..

[26] Banks N (1947). On some Acarina from North America. Psyche.

[27] Sellnick, M. Die Oribatiden der Bernsteinsammlung der Universität Königsberg. *Schr. physik.-ökonom. Ges. Königsberg* **59**, 21–42 (1931).

[28] Mihelčič, F. Beitrag zur Kenntnis der Oribatei und Collembolen der Humusböden. *Arch. Zool. Ital.* **37**, 93–106 (1952).

[29] Seniczak, A. & Seniczak, St. Morphological ontogeny of *Caleremaeus monilipes* (Acari: Oribatida: Caleremaeidae), with comments on *Caleremaeus* Berlese. *Syst. Appl. Acarol*. **24**, 1995–2009. 10.11158/saa.24.11.3 (2019).

[30] Ayyildiz N, Toluk A, Taskiran M, Tasdemir A (2011). Two new records of the genera *Cepheus* CL Koch, 1835 and *Caleremaeus* Berlese, 1910 (Acari: Oribatida) from Turkey, with notes on their distribution and ecology. Türk. entomol. bült..

[31] Karppinen E, Krivolutsky DA (1982). List of oribatid mites (Acarina, Oribatei) of northern palaearctic region I. Europe. Acta Entomol. Fenn..

[32] Luxton M (1996). Oribatid mites of the British Isles: A check-list and notes on biogeography (Acari: Oribatida). J. Nat. Hist..

[33] Weigmann, G. *Die Tierwelt Deutschlands*, 76. Teil Hornmilben (Oribatida), Goecke & Evers, Keltern (2006).

[34] Fischer BM, Schatz H (2013). Biodiversity of oribatid mites (Acari: Oribatida) along an altitudinal gradient in the Central Alps. Zootaxa.

[35] Höpperger M, Schatz H (2013). Hornmilben (Acari, Oribatida) von Castelfeder (Südtirol, Italien). Gredleriana.

[36] Schuster R (1997). Erstnachweise einiger bodenbewohnender Hornmilben-Arten für das Bundesland Oberösterreich (Acari, Oribatida). Beitr. Naturk. Oberösterreichs.

[37] Schatz, H. *U.-Ordn. Oribatei, Hornmilben. Catalogus Faunae Austriae*, Teil IXi. Österreichische Akademie der Wissenschaften, Wien (1983).

[38] Schatz, H. Ökologische Untersuchungen an Wirbellosen des zentralalpinen Hochgebirges (Obergurgl, Tirol). - II. Phänologie und Zönotik von Oribatiden (Acari). *Alpin-Biol. Stud., v. 10* (ed Janetschek, H.) 15–120 (Veröff. Univ. Innsbruck, v. 117, 1979).

[39] Lienhard A, Schaeffer S, Krisper G, Sturmbauer C (2014). Reverse evolution and cryptic diversity in putative sister families of the Oribatida (Acari). J. Zool. Syst. Evol. Res..

[40] Carstens BC, Pelletier TA, Reid NM, Satler JD (2013). How to fail at species delimitation. Mol. Ecol..

[41] Mąkol J, Saboori A, Felska M (2019). Inter-and intraspecific variability of morphological and molecular characters in *Allothrombium* species, with special reference to *Allothrombium fuliginosum*. Exp. Appl. Acarol..

[42] Hebert, P. D., Stoeckle, M. Y., Zemlak, T. S. & Francis, C. M. Identification of birds through DNA barcodes. *PLoS biology* **2**(10), e312 (2004). 10.1371/journal.pbio.0020312 PMC518999 15455034

[43] Salomone N, Emerson BC, Hewitt GM, Bernini F (2002). Phylogenetic relationships among the Canary Island Steganacaridae (Acari, Oribatida) inferred from mitochondrial DNA sequence data. Mol. Ecol..

[44] Delić T, Trontelj P, Rendoš M, Fišer C (2017). The importance of naming cryptic species and the conservation of endemic subterranean amphipods. Sci. Rep..

[45] Fontaneto D, Kaya M, Herniou EA, Barraclough TG (2009). Extreme levels of hidden diversity in microscopic animals (Rotifera) revealed by DNA taxonomy. Mol. Phylogenet. Evol..

[46] Laumann M (2007). Speciation in the parthenogenetic oribatid mite genus *Tectocepheus* (Acari, Oribatida) as indicated by molecular phylogeny. Pedobiologia.

[47] Martin P, Dabert M, Dabert J (2010). Molecular evidence for species separation in the water mite *Hygrobates nigromaculatus* Lebert, 1879 (Acari, Hydrachnidia): Evolutionary consequences of the loss of larval parasitism. Aquat. Sci..

[48] Zhang B, Chen TW, Mateos E, Scheu S, Schaefer I (2018). Cryptic species in *Lepidocyrtus lanuginosus* (Collembola: Entomobryidae) are sorted by habitat type. Pedobiologia.

[49] Luxton M (1972). Studies on the oribatid mites of a Danish beech wood soil I. Nutritional biology. Pedobiologia.

[50] Skubala, P. & Maslak, M. Succession of oribatid fauna (Acari, Oribatida) in fallen spruce trees: Deadwood promotes species and functional diversity in *Trends in acarology*: *Proceedings of the 12*^*th*^* International Congress* (eds Sabelis, M. W. & Bruin, J.) 123–128 (Springer, Dordrecht, 2009).

[51] Siira-Pietikäinen A, Penttinen R, Huhta V (2008). Oribatid mites (Acari: Oribatida) in boreal forest floor and decaying wood. Pedobiologia.

[52] Skubała P, Sokołowska M (2006). Oribatid fauna (Acari, Oribatida) in fallen spruce trees in the Babia Góra National Park. Biol Lett..

[53] Fontaneto D, Barraclough TG, Chen K, Ricci C, Herniou EA (2008). Molecular evidence for broad-scale distributions in bdelloid rotifers: Everything is not everywhere but most things are very widespread. Mol. Ecol..

[54] Lindo, Z. Communities of Oribatida associated with litter input in western red cedar tree crowns: Are moss mats ‘magic carpets’ for oribatid mite dispersal? in *Trends in Acarology Proceedings of the 12th International Congress* (eds Sabelis, M. W. & Bruin, J.) 143–148 (Springer, Dordrecht, 2009).

[55] Lehmitz R, Russell D, Hohberg K, Christian A, Xylander WE (2011). Wind dispersal of oribatid mites as a mode of migration. Pedobiologia.

[56] Krivolutsky DA, Lebedeva NV (2004). Oribatid mites (Oribatei) in bird feathers: Passeriformes. Acta Zool. Lituanica.

[57] Lebedeva, N. V. & Lebedev, V. D. Transport of oribatid mites to the polar areas by birds in *Integrative Acarology. Proceedings of the 6th European Congress of the European Association of Acarologists* (eds Bertrand, M. *et al*.) 359–367 (2008).

[58] Lebedeva NV, Poltavskaya MP (2013). Oribatid mites (Acari, Oribatida) of plain area of the Southern European Russia. Zootaxa.

[59] Manu M (2019). Soil mite communities (Acari: Mesostigmata, Oribatida) as bioindicators for environmental conditions from polluted soils. Sci. Rep..

[60] Zaitsev AS, Gongalsky KB, Persson T, Bengtsson J (2014). Connectivity of litter islands remaining after a fire and unburnt forest determines the recovery of soil fauna. Appl. Soil Ecol..

[61] Moraza ML, Peña MA (2005). Oribatid mites (Acari: Oribatida) in selected habitats of La Gomera (Canary Islands, Spain). Boln. Asoc. Esp. Ent..

[62] Miko, L. & Travé, J. Hungarobelbidae n. Fa., with a description of *Hungarobelba pyrenaica* n. sp. (Acarina, Oribatida). *Acarologia* **37**, 133–155 (1996).

[63] Subías, L. S. & Arillo, A. Acari, Oribatei, Gymnonota II. Oppioidea in *Fauna Iberica*, *vol. 15* (eds Ramos A. *et al.*) (Madrid, Museo de Ciencias Naturales, 2001).

[64] Bulanova-Zachvatkina, E. M. Family Caleremaeidae in *A key to the soil-inhabiting mites*. *Sarcoptiformes* (eds Gilyarov M. S. & Krivolutsky D. A.) 193–194 (Moscow: Nauka, 1975) [in Russian]

[65] Heethoff, M. Cryptic species—Conceptual or terminological chaos? A response to Struck et al. *Trend Ecol. Ecol.* **33** (5), 310 (2008). 10.1016/j.tree.2018.02.006 29605087

[66] Struck (2018). Cryptic species–More than terminological Chaos: A reply to Heethoff. Trend Ecol..

[67] Darwin, C. R. On the origin of species by means of natural selection, or the preservation of favoured races in the struggle for life. (London, John Murray, 1859). PMC5184128 30164232

[68] Lienhard, A. & Schaeffer, S. Extracting the invisible: Obtaining high quality DNA is a challenging task in small arthropods. *PeerJ.* **7**, e6753 (2019). 10.7717/peerj.6753 PMC6463856 30997294

[69] Otto, J. C. & Wilson, K. Assessment of the usefulness of ribosomal 18S and mitochondrial COI sequences in Prostigmata phylogeny in *Acarology: Proceedings of the 10th International Congress* (eds. Halliday R. B. *et al.*) 100–109 (CSIRO Publishing, 2001).

[70] Regier JC, Shultz JW (1997). Molecular phylogeny of the major arthropod groups indicates polyphyly of crustaceans and a new hypothesis for the origin of hexapods. Mol. Biol. Evol..

[71] Schaeffer, S., Krisper, G., Pfingstl, T. & Sturmbauer, C. Description of *Scutovertex pileatus* sp. nov. (Acari, Oribatida, Scutoverticidae) and molecular phylogenetic investigation of congeneric species in Austria. *Zool. Anz.* **247**, 249–258 (2008).

[72] Kumar, S., Stecher, G. & Tamura, K. MEGA7: Molecular evolutionary genetics analysis version 7.0 for bigger datasets. *Mol. Biol. Evol.* **33**, 1870–1874 (2016). 10.1093/molbev/msw054 PMC8210823 27004904

[73] Lefort V, Longueville JE, Gascuel O (2017). SMS: smart model selection in PhyML. Mol. Biol. Evol..

[74] Guindon, S. *et al*. New algorithms and methods to estimate maximum-likelihood phylogenies: assessing the performance of PhyML 3.0. *Syst. Boil.* **59**(3), 307–321 (2010). 10.1093/sysbio/syq010 20525638

[75] Ronquist F, Huelsenbeck JP (2003). MrBayes 3: Bayesian phylogenetic inference under mixed models. Bioinformatics.

[76] Rambaut, A., Drummond, A. J., Xie, D., Baele, G. & Suchard, M. A. Posterior summarisation in Bayesian phylogenetics using Tracer 1.7. *Syst. Biol*. **67**(5), 901–904. 10.1093/sysbio/syy032 (2018). 10.1093/sysbio/syy032 PMC6101584 29718447

[77] Clement M, Snell Q, Walker P, Posada D, Crandall K (2002). TCS: Estimating gene genealogies. Parallel Distrib. Process. Symp. Int. Proc..

[78] Leigh JW, Bryant D (2015). popart: full-feature software for haplotype network construction. Methods Ecol. Evol..

[79] Miller MP (2005). Alleles in Space (AIS): computer software for the joint analysis of interindividual spatial and genetic information. J. Hered..

[80] Pons J (2006). Sequence-based species delimitation for the DNA taxonomy of undescribed insects. Syst. Biol..

[81] Zhang J, Kapli P, Pavlidis P, Stamatakis A (2013). A general species delimitation method with applications to phylogenetic placements. Bioinformatics.

[82] Puillandre N, Lambert A, Brouillet S, Achaz G (2012). ABGD, Automatic Barcode Gap Discovery for primary species delimitation. Mol. Ecol..

[83] Kapli P (2017). Multi-rate Poisson tree processes for single-locus species delimitation under maximum likelihood and Markov chain Monte Carlo. Bioinformatics.

[84] Rannala B, Yang Z (2003). Bayes estimation of species divergence times and ancestral population sizes using DNA sequences from multiple loci. Genetics.

[85] Yang Z, Rannala B (2010). Bayesian species delimitation using multilocus sequence data. Proc. Natl. Acad. Sci..

