# Author Correction: Hidden biodiversity in microarthropods (Acari, Oribatida, Eremaeoidea, Caleremaeus)

**DOI:** 10.1038/s41598-022-05214-x

**Published:** 2022-01-11

**Authors:** Andrea Lienhard, Günther Krisper

**Affiliations:** Institute of Biology, University of Graz, Universitätsplatz 2, 8010 Graz, Austria

Correction to: *Scientific Reports* https://doi.org/10.1038/s41598-021-02602-7, published online 30 November 2021

The original version of this Article contains errors.

The original version of this Article does not include evidence of registration in ZooBank within the published Article itself, as required by Article 8.5.3 of the International Code of Zoological Nomenclature [1]. Therefore, the details of the newly proposed species names *Caleremaeus alpinus, Caleremaeus elevates, Caleremaeus hispanicus, Caleremaeus lignophilus* and *Caleremaeus mentobellus* and a description of the Nomenclatural Acts were not available at the time of publication.

This published work and the Nomenclatural Acts it contains have been registered in ZooBank with the LSID urn:lsid:zoobank.org:pub:B6DE6ABE-9F85-4024-A389-F3599935867F for this publication and the following for the new species:

*Caleremaeus alpinus*:

urn:lsid:zoobank.org:act:066DEBE4-D87A-4C0C-9662-4D5ED3C54909

*Caleremaeus elevatus*:

urn:lsid:zoobank.org:act:53F3A9EA-A36E-4260-90C4-888F7AA9632F

*Caleremaeus hispanicus*:

urn:lsid:zoobank.org:act:4923844B-F547-4ACF-86D6-A5CA5F3A010D

*Caleremaeus lignophilus*:

urn:lsid:zoobank.org:act:91C9BCD8-CE2A-4F1F-AADC-72C4498AD6B9

*Caleremaeus mentobellus*:

urn:lsid:zoobank.org:act:1AA1DC07-62B0-4EFB-8FB2-6096182506E6
