# Supplementary material for: Hidden biodiversity in microarthropods (Acari, Oribatida, Eremaeoidea, Caleremaeus)
Source: Sci Rep. 2021 Nov 30;11:23123. doi: 10.1038/s41598-021-02602-7 (PMC8632897; doi:10.1038/s41598-021-02602-7)
Supplement: Supplementary file 1 — Supplementary Information. [file 41598_2021_2602_MOESM1_ESM.pdf]

Supplementary Information

Hidden biodiversity in microarthropods (Acari, Oribatida, Eremaeoidea, Caleremaeus)

Andrea Lienhard[1] & Günther Krisper[1]*

[1] University of Graz, Institute of Biology, Universitätsplatz 2, 8010 Graz, Austria.
*guenther.krisper@uni-graz.at

Molecular phylogenetic analyses (part 1)

Phylogenetic analyses such as Neighbor joining (NJ) and maximum parsimony (MP) were performed using PAUP* version 4.02a [1]. These topologies were statistically supported by bootstrap values (BT; 5,000 replicates for NJ, 1,500 for MP and 1,000 for ML). Using the program MODELTEST version 3.06 [2] the TrN+I+G model was selected for NJ and MP analyses. Maximum Parsimony was performed using a heuristic tree search, random addition of sequences (1,000 replicates) and the TBR branch swapping algorithm (2,000 replicates). Uninformative characters were excluded from all analyses and all characters were considered with equal weights. Maximum likelihood (ML) trees were conducted in RAxML-7.0.3-WIN [3]. The constructed trees were combined in a 50 % majority rule consensus tree by means of the program PAUP*. To assess whether the topologies obtained by four different tree building algorithms differ significantly, a SH-test [4] was performed as implemented in PAUP*.
(1) GMYC was conducted by means of the splits package as implemented in R version 3.3.2 [5], http://r-forge.r-project.org/). The steps for GMYC analyses were conducted as recommended by Talavera *et al.* [6]. Cadence v 1.0.1 [7] was used to evaluate substitution rate differences for COI and EF-1α dataset. Distribution of branch length was plotted for every individual for each tree. There were no considerable variations in the relative rates among ingroup taxa, therefore a strict molecular clock was applied for both gene fragments. For the GMYC analysis ultrametric input trees are required, therefore a combined ultrametric tree was created (MCMC simulation with 200 million generations, sampled every 1000th generation, Yule tree model, a clock rate of 2.15 for COI, and an estimated rate for EF-1a was entered) by means of the program BEAUti as implemented in BEAST v1.8.3 [8]. TRACER v.1.7.1 was again used to verify the chains had reached stationarity. The remaining post burn-in trees were combined with TreeAnnotator 1.8.3 (also implemented in the BEAST package, settings after Casquet *et al.* [9], posterior probability limit of 0.5). A single-threshold was first employed due to its better performance in delimitation [10], however, the multiple-threshold analysis showed same results. (2) The bPTP method is an updated version of the original maximum likelihood PTP and adds Bayesian support values to delimited species on the input tree. bPTP analyses require a bifurcated phylogenetic

tree (not an ultrametric tree like the GMYC model). Therefore, the BI of the concatenated dataset (tree settings mentioned above) were uploaded to the web server (https://species.h-its.org/ptp/) applying 500,000 MCMC generations. The removal of the outgroup had no impact on species delimitation results of the ingroup. (3) The ABGD Barcoding gap analysis was conducted with the COI dataset and default settings (simple distance and K2P gave the same results) via the ABGD web server (http://wwwabi.snv.jussieu.fr/public/abgd/abgdweb.html). ABGD is an automatic procedure that sorts the sequences into putative species based on distance. The ABGD is a non-tree-based method and only requires an alignment file. (4) For mPTP, a BI tree in Newick format (same as for bPTP, from the BI analysis with branch length) was submitted to the mPTP web server (https://mptp.h-its.org/#/tree). The multi rate poisson tree processes method was selected. (5) BPP, which is a multi-locus species delimitation analysis was conducted by means of the program bppX (version 1.2.2), which is the graphical interface of the bpp program (http://abacus.gene.ucl.ac.uk/software.html). To confirm the consistency between runs, we performed five independent runs. We also used different combinations of priors for ancestral population size (θ) and root age (τ_0) as recommended by Leaché & Fujita [11] and Yang & Rannala [12], because of the fact that these parameters can affect the posterior probabilities for models. The obtained posterior probability values showed consistency between runs and among different prior settings. Prior five clades (and two outgroup clades) were defined and confirmed as delimited species. Except of the ABGD method (COI dataset) the concatenated dataset was analysed for species delimitation analyses. The use of multiple genes (which are unlinked) by concatenation of data present a more adequate approach in species delimitation, although nuclear genes evolve more slowly and therefore the statistical support or the resolution of revealed clades may be lower [13].

Supplementary references – part 1

1. Swofford, D.L. PAUP* Phylogenetic Analysis Using Parsimony (*and Other Methods), version 4.02a. (Sinauer, Sunderland, MA, USA 2002).

2. Posada, D. & Crandal, K. MODELTEST: testing the model of DNA substitution. *Bioinformatics* **14**, 817–818 (1998).

3. Stamatakis, A. RAxML-VI-HPC: maximum Likelihood-based phylogenetic analyses with thousands of taxa and mixed models. *Bioinformatics* **22,** 2688–2690 (2006).

4. Shimodaira, H. & Hasegawa, M. Multiple comparison of loglikelihoods with applications to phylogenetic inference. *Mol. Biol. Evol.* **16,** 1114–1116 (1999).

5. R Core Team. R: A language and environment for statistical computing. R Foundation for Statistical Computing, Vienna, Austria. URL http://www.R-project.org/ (2013).

6. Talavera, G., Dincă, V. & Vila, R. Factors affecting species delimitations with the GMYC model: insights from a butterfly survey. *Methods Ecol. Evol.* **4**(12), 1101–1110 (2013).

7. Wilcox, T. P., García de León, F. J., Hendrickson, D. A. & Hillis, D. M. Convergence among cave catfishes: long-branch attraction and a Bayesian relative rate test. *Mol. Phylogenet. Evol.* **31**, 1101–1113 (2004).

8. Drummond, A. J., Suchard, M. A., Xie, D. & Rambaut, A. Bayesian phylogenetics with BEAUti and the Beast 1.7. *Mol. Biol. Evol.* **29**, 1969–1973 (2012).

9. Casquet *et al.* Community assembly on remote islands: a comparison of Hawaiian and Mascarene spiders. *J. Biogeogr.* **42**, 39–50 (2015).

10. Fujisawa, T. & Barraclough, T. G. Delimiting species using single-locus data and the Generalized Mixed Yule Coalescent approach: a revised method and evaluation on simulated data sets. *Syst. Biol.* **62**, 707–724 (2013).

11. Leaché, A. D. & Fujita, M. K. Bayesian species delimitation in West African forest geckos (*Hemidactylus fasciatus*). *Proc. Royal Soc. B.* **277**, 3071–3077 (2010).

12. Yang, Z. & Rannala, B. Bayesian species delimitation using multilocus sequence data. *PNAS* **107**(20), 9264–9269 (2010).

13. Lukhtanov, V. A. Species delimitation and analysis of cryptic species diversity in the XXI century. *Entomol. Rev.* **99**(4), 463–472 (2019).

14. Lienhard, A., Schaeffer, S., Krisper, G. & Sturmbauer, C. Reverse evolution and cryptic diversity in putative sister families of the Oribatida (Acari). *J. Zool. Syst. Evol. Res.* **52**, 86–93 (2014).

Supplementary figures and tables - part 1

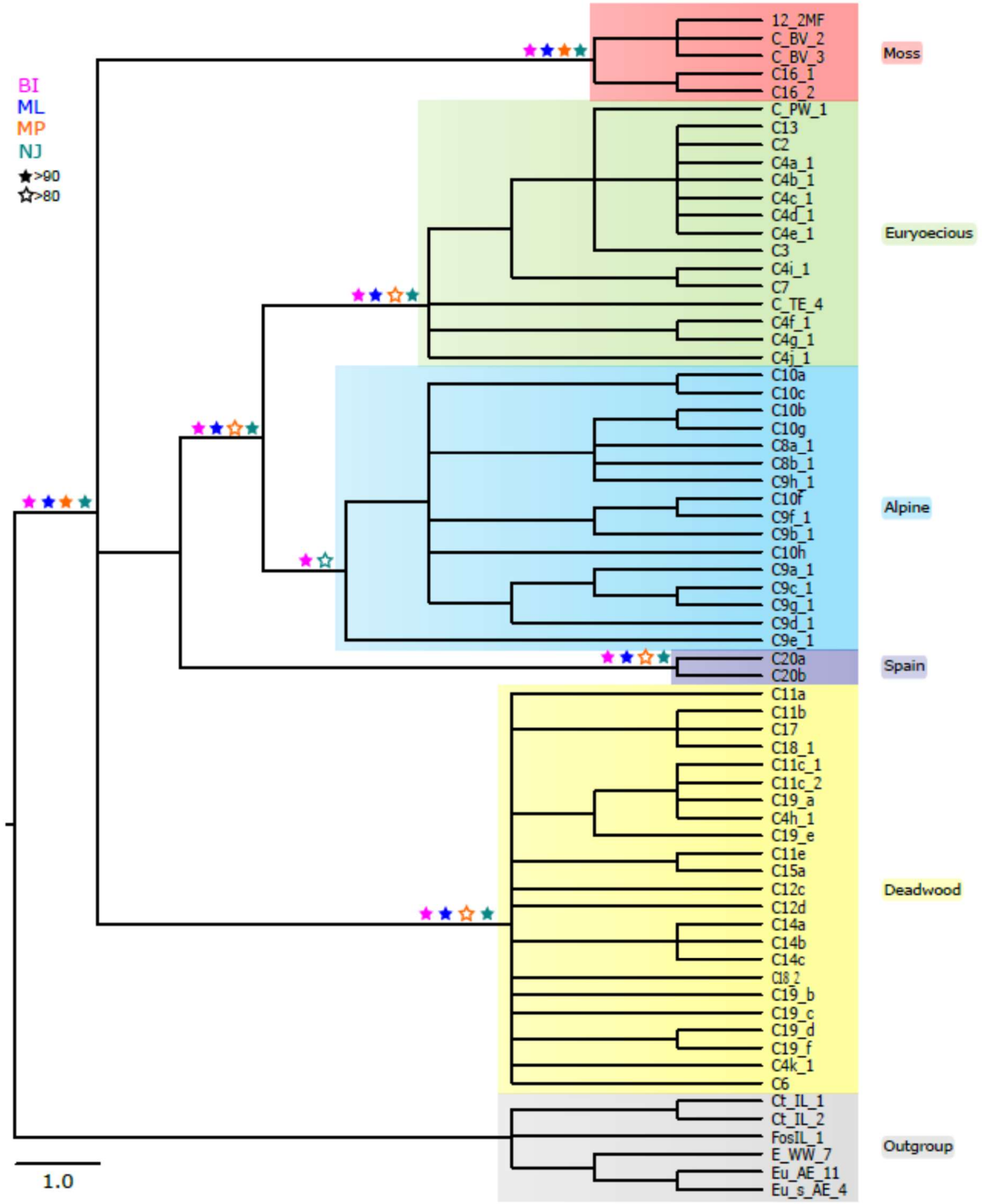

Fig. S1 75 % Majority rule consensus tree comprising four tree building methods (BI, ML, MP and NJ) for *Caleremaeus* specimens based on the COI dataset. Posterior probabilities for BI analysis and bootstrap values for ML, MP and NJ are indicated by stars near nodes and are given for main ingroup nodes. Filled stars show values >90 and framed ones values >80. Colored boxes show species clades and correspond to those in Figure 1. Ct = *Ctenobelba*, Fos = *Fosseremus*, E = *Eremaeus*, Eu = *Eueremaeus*

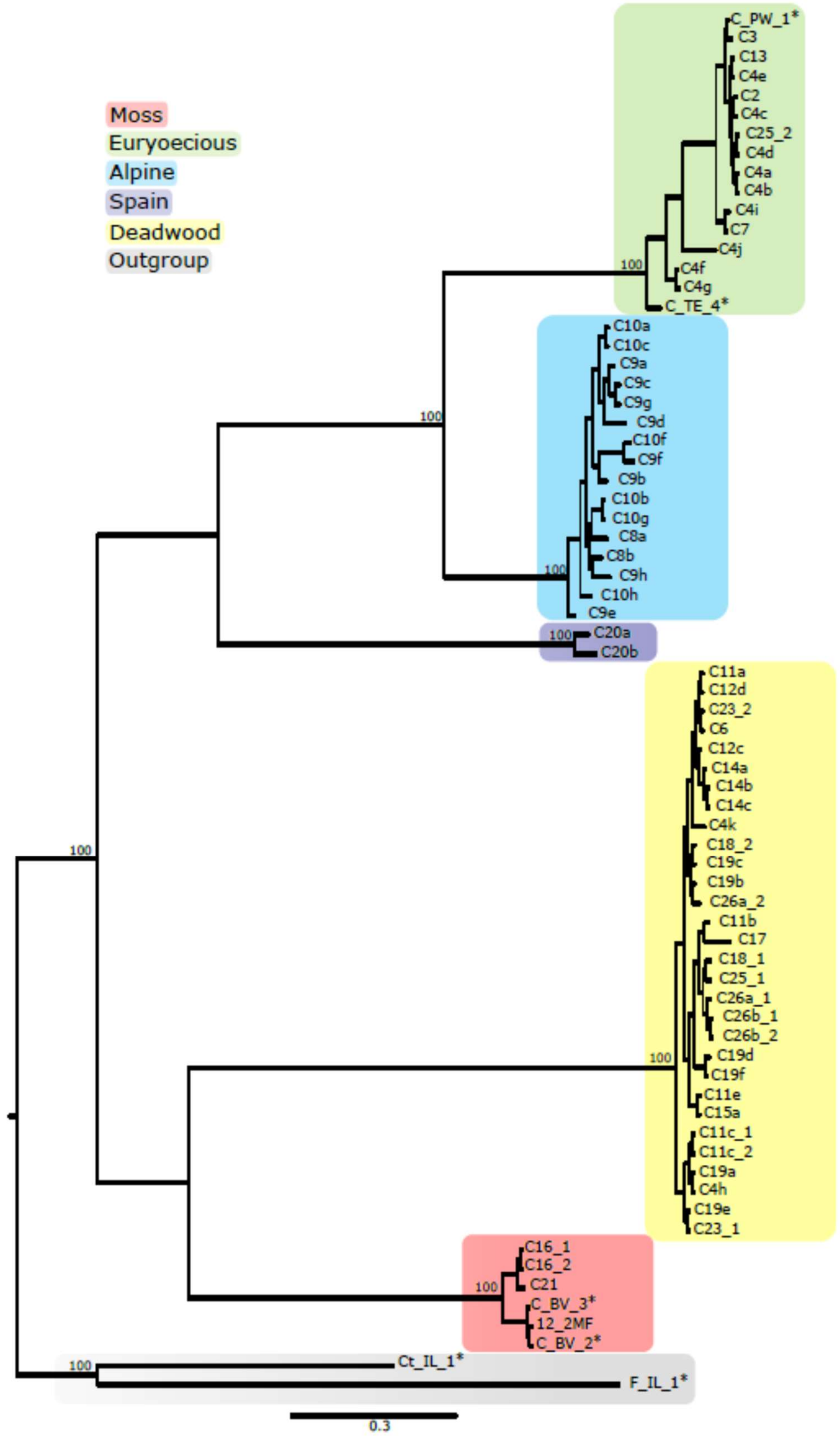

Fig. S2 Bayesian inference tree based on the COI dataset comprising 72 *Caleremaeus* specimens. Posterior probabilities >95 for main nodes are shown near nodes. Colored boxes show species clades and correspond to those in Figure 1.

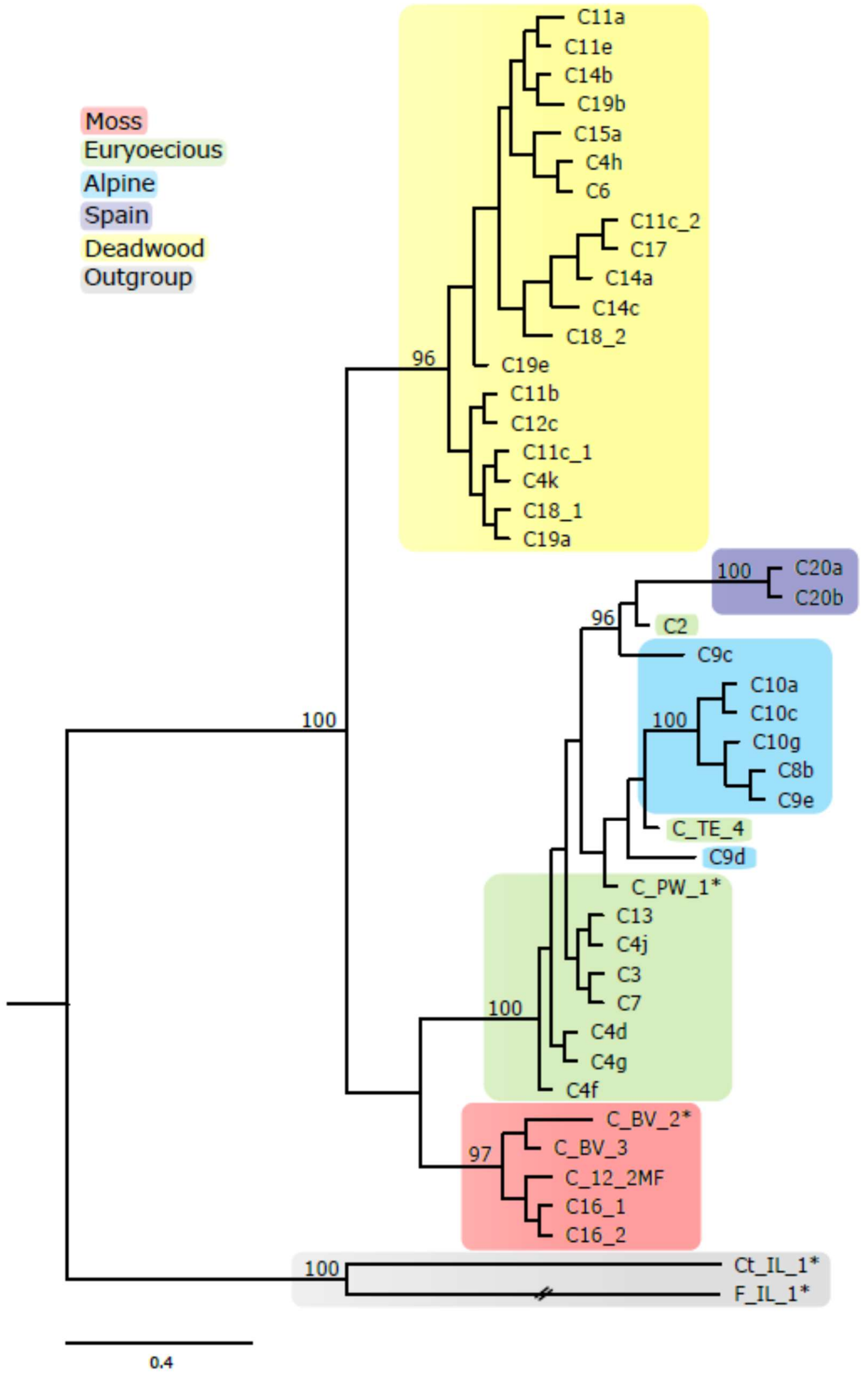

Fig. S3 Bayesian inference tree based on the EF-1α dataset comprising 45 *Caleremaeus* specimens. Posterior probabilities >95 for main nodes are shown near nodes. Colored boxes show species clades and correspond to those in Figure 1.

**SH test**			
Tree	-ln L	Diff.-ln L	P
NJ	1387.57967	0.00000	0.9990
MP	1387.57967	0.00000	0.9115
ML	1403.98305	16.40339	0.1140
BI	1387.57967	-	-

Table S1. Comparison of four different phylogenetic tree-building methods (NJ, BI, MP, ML) for the COI dataset.

	euryoecious (10)	alpine (7)	Spain (2)	deadwood (19)	moss (5)
euryoecious (16)	***1.85*** **/** 0.04	0.86	1.76	3.30	2.50
alpine (16)	***13.62***	***1.74*** **/** 0.90	2.54	4.12	3.33
Spain (2)	***20.45***	***18.48***	***2.12*** **/** 0.00	4.07	3.60
deadwood (30)	***22.29***	***21.22***	***21.82***	***0.83*** **/** 0.29	3.18
moss (6)	***20.47***	***19.60***	***19.79***	***20.57***	***1.39*** / 0.36

Table S2. Averaged intra- and interclade uncorrected p-distances in %, measured for the COI gene fragment (bold) and for the EF-1α fragment. Number of investigated specimens per clade is given in parenthesis.

Sampling locality	Date	Coordinates	Altitude [m asl]	Sample	Sample source	Vegetation/Surrounding	Species identification (genetics; *morphology*)	Clade
Terz/Schöpfelspitz (St)	14.07.2010	47.767215, 15.429272	950	Mosses and lichens	B of *Fagus sylvatica*	Open forest with free-standing *Fagus sylvatica*	CM_TE_4	"euryoecious"
Peggau (St)	21.07.2010	47.207533, 15.348916	500-540	Mosses	G	Mixed forest (*Carpinus betulus, Fagus sylvatica, Tilia* sp.)	CM_PW_1	"euryoecious"
Bad Vöslau (L)	30.07.2010	47.969167, 16.205278	300-400	Mosses	B of *Pinus nigra*	Pine forest (*Pinus nigra, Quercus petraea, Carpinus betulus, Fagus sylvatica)*	CM_BV_2-3	"moss"
	13.08.2014			Deadwood	dt		C_15a	"deadwood"
Weißenkirchen in der Wachau (L)	20.08.2010	48.404183, 15.443666	700	Mosses	RB	Mixed forest (*Quercus* sp., *Carpinus betulus, Fagus sylvatica, Larix decidua*)	12_2_MF	"moss"
Mantscha/Graz (St)	16.04.2014	47.035389, 15.364500	424	Mosses	*B of Alnus* sp.	Mixed forest (*Carpinus betulus, Fagus sylvatica*)	C2	"euryoecious"
		47.030572, 15.367435	445	Mosses	RB		C3	"euryoecious"
	20.05.2014	47.035833, 15.364639	424	Deadwood, mosses	dt (*Alnus* sp.)		C6	"deadwood"
		47.038674, 15.367197	460	Deadwood, moss	dt		C12c	"deadwood"
				Moss, deadwood	dt, G		C12d	"deadwood"
Weizklamm (St)	24.04.2014	47.270828, 15.584375	791	Lichens	RB	Mixed deciduous forest *(Ostrya carpinifolia)*	C4a	"euryoecious"
				Mosses	RB		C4b; *1 PT a*	
		47.270583, 15.584056	784	Mosses	B (*Ostrya carpinifolia*)		C4c	
		47.269960, 15.583504		Mosses	bB of *Fagus sylvatica*		C4d; *HTyp+2 PT a1, PT b*	
		47.267731, 15.584458	694	Mosses	RB		C4e	
		47.268417, 15.583944	700	Mosses	G	*Sesleria albicans* lawn	C4f	
				Soil and litter	G		C4g	
		47.268778, 15.584056	715	Deadwood	dt		C4h	"deadwood"

Sampling locality	Date	Coordinates	Altitude [m asl]	Sample	Sample source	Vegetation/Surrounding	Species identification (genetics; *morphology*)	Clade
		47.270083, 15.583444	730	Mosses	RB	Mixed deciduous forest *(Ostrya carpinifolia)*	C4i; *1 PT b*	"euryoecious"
		47.269943, 15.583446	700	Mosses	RB		C4j	
		47.271028, 15.580278	686	Deadwood, mosses	dt		C4k	"deadwood"
Deutschlandsberger Klause (St)	22.05.2014	46.810833, 15.199167	405	Mosses	RB	Mixed forest	C7	"euryoecious"
Hochschwab (St)	25.06.2014	47.607500, 15.205278	1830	Mosses	G	alpine meadow	C8a	"alpine"
	26.06.2014	47.602500, 15.187778	1500	Lichens	G	*Pinus mugo*	C8b	
Saualpe (C)	03.07.2014	46.922075, 14.677143	1900	Grass with soil	G	alpine meadow	C9a	"alpine"
		46.919217, 14.674637	1900	Mosses	RB		C9b	
		46.917144, 14.674149	1800	mosses	RB		C9c	
		46.899606, 14.663814	2024	moss	RB		C9d	
		46.897963, 14.662910	2030	Grass with soil	G		C9h	
		46.913389, 14.670306	1812	moss	RB	*Rhododendron* sp.	C9e	
		46.921272, 14.672294	1788	Deadwood	dt (*Larix* sp.)		C9f	
		46.919361, 14.674278	1914	Soil and litter	G	*Loiseleuria*	C9g	
Handalpe (St, C)	17.07.2014	46.844250, 15.019639	1811	Mosses	G	alpine meadow	C10a; *5 PTa*	"alpine"
				Mosses	G	Alpine meadow	C10b; *HTyp+3 PTa*	

Sampling locality	Date	Coordinates	Altitude [m asl]	Sample	Sample source	Vegetation/Surrounding	Species identification (genetics; *morphology*)	Clade
				Soil	G	*Loiseleuria*	C10c	
		46.846222, 15.019472	1851	Mosses	RB	Alpine meadow	C10f	
		46.844111, 15.022833	1824	Grass, moss, lichens, soil	G	Alpine meadow	C10g	
		46.844139, 15.022778	1822	Soil, grass	G	Alpine meadow	C10h	
Leechwald/Graz (St)	05.08.2014	47.084061, 15.462311	400-450	Deadwood	dt	Mixed deciduous forest	C11a	"deadwood"
		47.084324, 15.462496		Deadwood	dt		C11b; *HTyp+5 PTa, 5 PTb*	
		47.084182, 15.463189		Moss, deadwood	ts		C11c_1-2	
		47.086086, 15.464633		Deadwood	dt		C11e	
Stift Rein (St) leg. Kuess	04.08.2014	47.136167, 15.282206	~480	Soil and litter	G	Mixed deciduous forest	C13	"euryoecious"
Dreistetten (L)	13.08.2014	47.862519, 16.094503	528	Deadwood, moss	dt	Mixed forest	C14a	"deadwood"
				Mosses	RB		C14b	
				Deadwood	dt		C14c	
Burg Eltz (Germany, RP) leg. McCullough	21.08.2014	50.205190, 7.336211	130	Mosses	RB	Mixed forest	C16_1-2	"moss"
Heimschuh (St) leg. Baumann	24.08.2014	46.766684, 15.462798	360	Deadwood	ts	Mixed deciduous forest	C17	"deadwood"
Pontebba (Italy, FG) leg. Kuess	26.09.2014	46.500472, 13.293186		Deadwood	dt	Mixed deciduous forest	C18_1-2	"deadwood"
Hohentauern (St)	29.09.2014	47.437333, 14.427917	1740	Deadwood	dt	Shore of Scheibelsee	C19a	"deadwood"
			1750	Deadwood		Shrubs with *Pinus cembra*	C19b	

Sampling locality	Date	Coordinates	Altitude [m asl]	Sample	Sample source	Vegetation/Surrounding	Species identification (genetics; *morphology*)	Clade
		47.438250, 14.426278			dt (*Pinus cembra*)		C19c	
		47.436028, 14.425000	1740	Deadwood		Besides the lake	C19d	
		47.440472, 14.447528	1450	Deadwood	ts	Coniferous forest	C19e	
		47.438722, 14.452083	1350	Deadwood	ts		C19f	
Busmayor/Barjas (Spain, CL) leg. Guerra	19.10.2014	42.623611, -7.031389	1300	Mosses	RB	beech wood	C20a<br>C20b	"Spain"
Ramsau (St) leg. Schäffer	07.08.2015	47.422000, 13.627664	1200	Deadwood		Picea forest		"deadwood"
			1163	Bark	tree			"deadwood"
	08.08.2015	47.434998, 13.614533	1427	Deadwood				"deadwood"
Ruine Aggstein, Wachau (L) leg. Ebermann	11.05.2015	48.314864, 15.422689	498	Mosses, lichens	RB	rocks surrounded by mixed (deciduous) forest	C21	"moss"
Falkendorf bei Murau (St) leg. Schuster	08.1987<br>07.1988	47.098889, 13.992778	~1115	Mosses	wooden shingle roof	meadows	*RS-1457,*<br>*1245*	"moss"
Koralpe, Hühnerstütze (St) leg. Baumann	23.9.2010	46.481493, 14.585870	1980	*Loiseleuria procumbens* cushions	G	Alpine meadow		"alpine"
Dolní Dvořiště (Czech Republic) leg. Schuster	20.09.2015	48.669536, 14.447222	~600	Mosses, deadwood	ts	Mixed forest	C23_1-2	"deadwood"
Lučice (Croatia)	10.11.2015	45.420095, 14.747139	764	Mosses (deadwood)	ts	*Abies* forest	C25_1<br>C25_2	"deadwood"<br>"euryoecious"
Lividraga (Croatia)	10.11.2015	45.483003, 14.592227	949	Deadwood	ts	*Picea* forest	C26a_1-2	"deadwood"
				Mosses	RB		C26b_1-2	"deadwood"
Lungau (S) leg. Schuster	25.10.2015	47.186561, 13.412729	1480	Mosses, deadwood	G	Coniferous forest	-	"deadwood"

Sampling locality	Date	Coordinates	Altitude [m asl]	Sample	Sample source	Vegetation/Surrounding	Species identification (genetics; *morphology*)	Clade
Bad Ischl (U) leg. Pfingstl	06.2014	47.703695, 13.614718	~470	Mosses	RB	-	-	"moss"
Strobl (S) leg. Reineke	23.05.2016	47.659907, 13.463337	740	Mosses	RB	-	-	"euryoecious"
Klafferbachtal, Hochficht (U)	19.07.1983	48.432384, 13.534132	752	Soil and litter	G	Mixed forest	-	"euryoecious"
Tamischbachgraben near Gstatterboden (St)	15.09.2002	47.360776, 14.391517	778	Deadwood	G	Mixed forest	-	"deadwood"

Table S3. List of investigated specimens with sampling locality, date, altitude, vegetation type, type of the samples and investigated species for genetic and morphological analyses. bB = on base of boles, B = on boles in 1m height, G = on ground, RB = on rocks and boulders, Ro = on rootstocks, dt = dead tree, ts = tree stump; C = Carinthia, L = Lower Austria, U = Upper Austria, S = Salzburg, St = Styria; RP = Rhineland-Palatinate; FG = Friuli–Venezia Giulia; CL = Castile and León.

			GenBank Accession Number	
**ID**	**Clade**	**Species**	**COI**	**EF-1α**
12_2MF	moss	*Caleremaeus elevatus* sp. nov.	OK545907	OK545973
C_BV_2	moss	*Caleremaeus* sp.	KF199430*	KF199366*
C_BV_3	moss	*Caleremaeus* sp.	KF199431*	-
C_PW_1	euryoecious	*Caleremaeus* sp.	KF199429*	KF199365*
C_TE_4	euryoecious	*Caleremaeus* sp.	KF199432*	-
C10a	alpine	*Caleremaeus alpinus* sp. nov.	OK545908	OK545974
C10b	alpine	*Caleremaeus alpinus* sp. nov.	OK545909	-
C10c	alpine	*Caleremaeus alpinus* sp. nov.	OK545910	OK545975
C10f	alpine	*Caleremaeus alpinus* sp. nov.	OK545911	-
C10g	alpine	*Caleremaeus alpinus* sp. nov.	OK545912	OK545976
C10h	alpine	*Caleremaeus alpinus* sp. nov.	OK545913	-
C11a	deadwood	*Caleremaeus lignophilus* sp. nov.	OK545914	OK545977
C11b	deadwood	*Caleremaeus lignophilus* sp. nov.	OK545915	OK545978
C11c_1	deadwood	*Caleremaeus lignophilus* sp. nov.	OK545916	OK545979
C11c_2	deadwood	*Caleremaeus lignophilus* sp. nov.	OK545917	OK545980
C11e	deadwood	*Caleremaeus lignophilus* sp. nov.	OK545918	OK545981
C12c	deadwood	*Caleremaeus lignophilus* sp. nov.	OK545919	OK545982
C12d	deadwood	*Caleremaeus lignophilus* sp. nov.	OK545920	-
C13	euryoecious	*Caleremaeus mentobellus* sp. nov.	OK545921	OK545983
C14a	deadwood	*Caleremaeus lignophilus* sp. nov.	OK545922	OK545984
C14b	deadwood	*Caleremaeus lignophilus* sp. nov.	OK545923	OK545985
C14c	deadwood	*Caleremaeus lignophilus* sp. nov.	OK545924	OK545986
C15a	deadwood	*Caleremaeus lignophilus* sp. nov.	OK545925	OK545987
C16_1	moss	*Caleremaeus elevatus* sp. nov.	OK545926	OK545988
C16_2	moss	*Caleremaeus elevatus* sp. nov.	OK545927	OK545989
C17	deadwood	*Caleremaeus lignophilus* sp. nov.	OK545928	OK545990
C18_1	deadwood	*Caleremaeus lignophilus* sp. nov.	OK545929	OK545991
C18_2	deadwood	*Caleremaeus lignophilus* sp. nov.	OK545930	OK545992
C19a	deadwood	*Caleremaeus lignophilus* sp. nov.	OK545931	OK545993
C19b	deadwood	*Caleremaeus lignophilus* sp. nov.	OK545932	OK545994
C19c	deadwood	*Caleremaeus lignophilus* sp. nov.	OK545933	-
C19d	deadwood	*Caleremaeus lignophilus* sp. nov.	OK545934	-
C19e	deadwood	*Caleremaeus lignophilus* sp. nov.	OK545935	OK545995
C19f	deadwood	*Caleremaeus lignophilus* sp. nov.	OK545936	-
C2	euryoecious	*Caleremaeus mentobellus* sp. nov.	OK545937	OK545996
C20a	spain	*Caleremaeus hispanicus* sp. nov.	OK545938	OK545997
C20b	spain	*Caleremaeus hispanicus* sp. nov.	OK545939	OK545998
C21	moss	*Caleremaeus elevatus* sp. nov.	OK545940	-
C23_1	deadwood	*Caleremaeus lignophilus* sp. nov.	OK545941	-
C23_2	deadwood	*Caleremaeus lignophilus* sp. nov.	OK545942	-
C25_1	deadwood	*Caleremaeus lignophilus* sp. nov.	OK545943	-
C25_2	euryoecious	*Caleremaeus mentobellus* sp. nov.	OK545944	-
C26a_1	deadwood	*Caleremaeus lignophilus* sp. nov.	OK545945	-
C26a_2	deadwood	*Caleremaeus lignophilus* sp. nov.	OK545946	-
C26b_1	deadwood	*Caleremaeus lignophilus* sp. nov.	OK545947	-
C26b_2	deadwood	*Caleremaeus lignophilus* sp. nov.	OK545948	-
C3	euryoecious	*Caleremaeus mentobellus* sp. nov.	OK545949	OK545999

C4a	euryoecious	*Caleremaeus mentobellus* sp. nov.	OK545950	-
C4b	euryoecious	*Caleremaeus mentobellus* sp. nov.	OK545951	-
C4c	euryoecious	*Caleremaeus mentobellus* sp. nov.	OK545952	-
C4d	euryoecious	*Caleremaeus mentobellus* sp. nov.	OK545953	OK546000
C4e	euryoecious	*Caleremaeus mentobellus* sp. nov.	OK545954	-
C4f	euryoecious	*Caleremaeus mentobellus* sp. nov.	OK545955	OK546001
C4g	euryoecious	*Caleremaeus mentobellus* sp. nov.	OK545956	OK546002
C4h	deadwood	*Caleremaeus lignophilus* sp. nov.	OK545957	OK546003
C4i	euryoecious	*Caleremaeus mentobellus* sp. nov.	OK545958	-
C4j	euryoecious	*Caleremaeus mentobellus* sp. nov.	OK545959	OK546004
C4k	deadwood	*Caleremaeus lignophilus* sp. nov.	OK545960	OK546005
C6	deadwood	*Caleremaeus lignophilus* sp. nov.	OK545961	OK546006
C7	euryoecious	*Caleremaeus mentobellus* sp. nov.	OK545962	OK546007
C8a	alpine	*Caleremaeus alpinus* sp. nov.	OK545963	-
C8b	alpine	*Caleremaeus alpinus* sp. nov.	OK545964	OK546008
C9a	alpine	*Caleremaeus alpinus* sp. nov.	OK545965	-
C9b	alpine	*Caleremaeus alpinus* sp. nov.	OK545966	-
C9c	alpine	*Caleremaeus alpinus* sp. nov.	OK545967	OK546009
C9d	alpine	*Caleremaeus alpinus* sp. nov.	OK545968	OK546010
C9e	alpine	*Caleremaeus alpinus* sp. nov.	OK545969	OK546011
C9f	alpine	*Caleremaeus alpinus* sp. nov.	OK545970	-
C9g	alpine	*Caleremaeus alpinus* sp. nov.	OK545971	-
C9h	alpine	*Caleremaeus alpinus* sp. nov.	OK545972	-
Cp_IL_1	-	*Ctenobelba pectinigera*	KF199433*	KF199367*
Cp_IL_2	-	*Ctenobelba pectinigera*	KF199434*	KF199368*
Fl_IL_1	-	*Fosseremus laciniatus*	KF199435*	KF199369*
E_WW_7	-	*Eremaeus* sp.	KF199377*	-
Eu_AE_11	-	*Eueremaeus* sp.	KF199397*	-
Eu_s_AE_4	-	*Eueremaeus silvestris*	KF199393*	-

Table S4. GenBank accession numbers for COI and EF-1α sequences comprising all specimens included in genetic investigations. *Lienhard *et al.* [14]

Description of species and remarks (part 2)

All light microscopical observations were made with mites mounted in permanent slides. During the hardening of the embedding medium the specimens roll easily on one side because of the shape of the notogaster (in Michael's slides too), therefore most drawings show a body slightly tipped over. The abbreviations for morphological structures used in text and figure legends follow Norton & Behan-Pelletier [1].

Common characters of all species described below

A very detailed generic description was published recently by Norton & Behan-Pelletier [1]. But it seems necessary for the reader to give an overview and to summarize characters common to all the species described below and to avoid repetitions in the text of the species descriptions.

The typical appearance of the mite is shown in lateral aspect in S2Fig.1 (referring to *Caleremaeus mentobellus*). Colour brown. Body and legs covered with cerotegument which shows different microstructure in various regions, partially the body looks encrusted. Cuticle of prodorsum, anterior part of notogaster, and epimeral region foveate.

Prodorsum: Lamellae (= median ridges or median prodorsal costulae) of prodorsum slightly diverging with conspicuous cusps bearing thick and spinose lamellar setae (exemplarily shown as SEM-micrograph in S2Fig.2 (*C. lignophilus*)). Laterally two further diverging ridges (tutorium) with projecting tubercles distally and proximal end forming enatiophyses with lateral ridge originating from the base of bothridium (prodorsal enantiophysis *eA* (see arrow in S2Fig. 3)). Bothridium cup-like with lateral open border. Bothridial seta with distally flattened club, covered with small scales. Adaxial of bothridia one pair of inwards curved slightly barbate interlamellar setae (*in*) of medium length situated on subrectangular sclerotized and foveate areas, which bear an enantiophysis (*dt*) directed towards the anterior border of notogaster. Between these areas cuticle without foveae.

Lateral podosoma: Exobothridial seta (*ex*) short and thin (see S2Fig. 4 (*C. alpinus*)). Lateral enantiophysis (*eLa*, *eLp*) spans sejugal groove above acetabulum II and III; a small gland orifice present near base of tubercle *eLa* (S2Fig. 5). Pedotectum I developed, pedotectum II missing; discidium (*dis*) as cone-like extension of a lateral keel originating in the humeral region.

Notogaster: Clearly separated by a deep dorsosejugal furrow from prodorsum. Contour more or less elliptical in dorsal aspect. The bulges of the notogaster were described correctly by Michael [2], (p. 17) as follows: "Immediately behind the anterior margin there is a broad, rounded, transverse elevation, not reaching the lateral margin. Behind this is a deep, linear depression, and then the centre of the abdomen, until within a quarter of its length from the hind margin, is occupied by a domed lump, followed by a smaller one, which touches the hind margin." (S2Fig. 6). Cuticle smooth with transverse irregular row of foveae in the area posterior of the transverse anterior bulge; at both

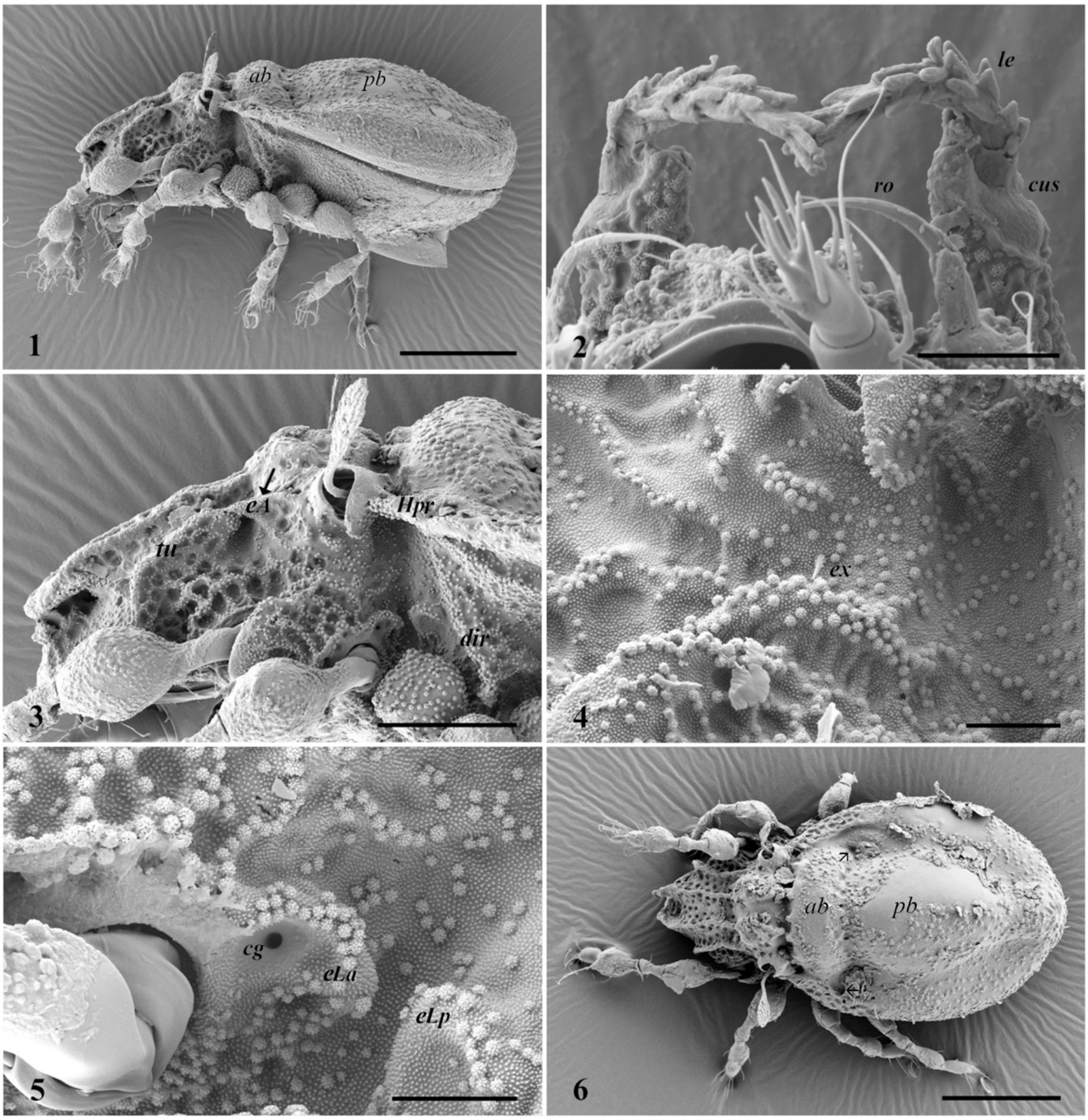

S2Figs 1-6. SEM micrographs. 1) *Caleremaeus mentobellus*, habitus lateral aspect; *ab* = transverse anterior bulge, *pb* = posterior longitudinal bulge. Scale bar = 100 µm. 2) *C. lignophilus*, ventral view of prodorsal cusps and spinose lamellar setae. Scale bar = 10 µm. 3) *C. mentobellus*, podosoma lateral aspect; prodorsal enantiophysis *eA* (see arrow). Scale bar = 50 µm. 4) *C. alpinus*, part of podosoma lateral aspect; cerotegument forming nodules and microtubercles, short exobothridial seta (*ex*). Scale bar = 10 µm. 5) *C. mentobellus*, part of podosoma lateral aspect (detail of S2Fig. 3); enantiophyses *eLa* and *eLp* crossing sejugal groove; posterior insertion of leg II, at base of *eLa* orifice of coxal gland *cg*. Scale bar = 10 µm. 6) *C. mentobellus,* dorsal view showing notogastral bulges; posterior to enantiophyses *Hpr* longitudinal zone with foveae forming a net-like structure. Arrows point to the steep cavities. Detaching cerotegument as artefact. Scale bar = 100 µm.

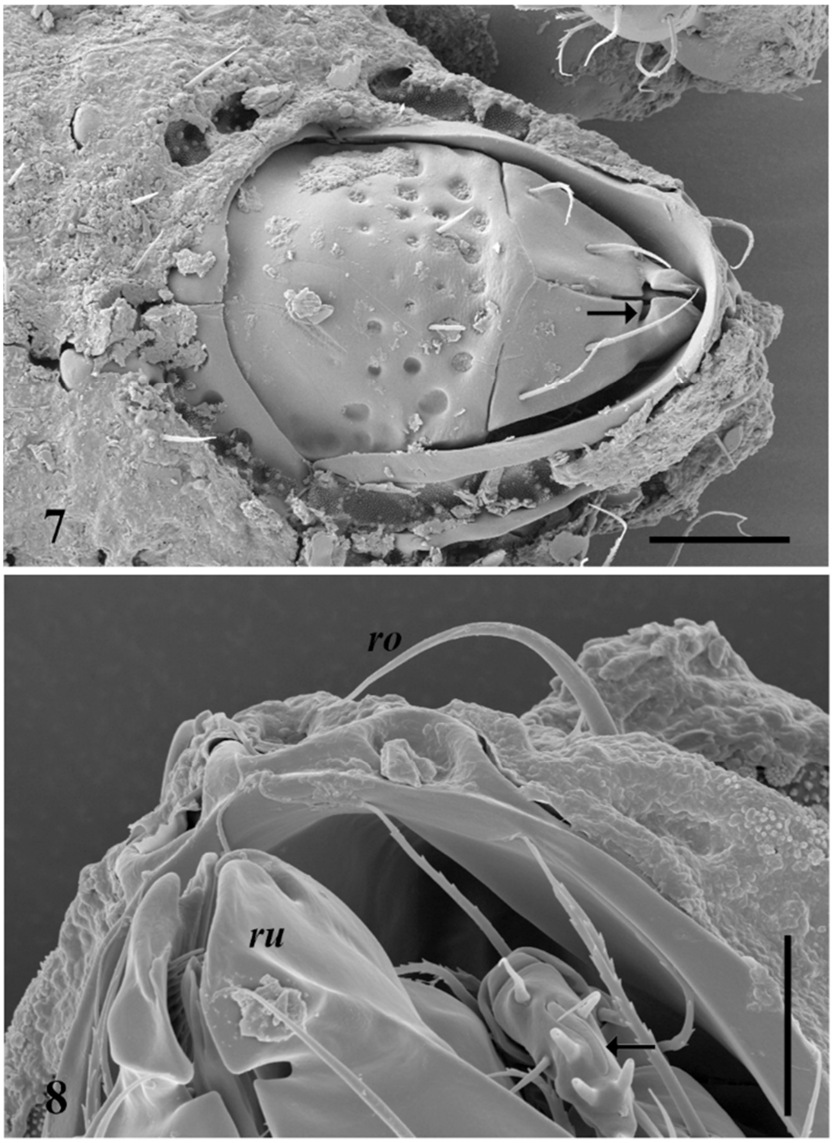

S2Figs. 7-8. *Caleremaeus mentobellus*: SEM micrographs. 7) ventral view of subcapitulum; rutella separated from genae by distinct incision (arrow), cuticle of both parts smooth. Scale bar = 20 µm. 8) ventral view of rostral region; palptarsus with recumbent tarsal solenidion (arrow). *ru* = rutellum, *ro* = rostral seta. Scale bar = 10 µm.

ends one steep cavity (S2Fig. 6). Anterior border of notogaster with rounded tubercles, their number underlying individual variation. One pair of more or less acute humeral enantiophyses (*Hpr*), their shape slightly varying; posterior of each humeral enantiophysis a narrow longitudinal zone with foveae forming a net-like structure almost reaching notogastral seta *la*. Ten pairs of notogastral setae (*c, la, lm, lp*, h_3, h_2, h_1, p_1, p_2, p_3) arranged peripherally; all of medium length and slightly barbed. Seta *c* on basis of humeral enantiophysis, h_1 longer and thicker than others. Only the lyrifissures *ia* and *im* are visible easily, *ip* posterior to h_1, *ips* and *ih* near lateral border of notogaster. Orifice of lateroopisthosomal gland (*gla*) present.

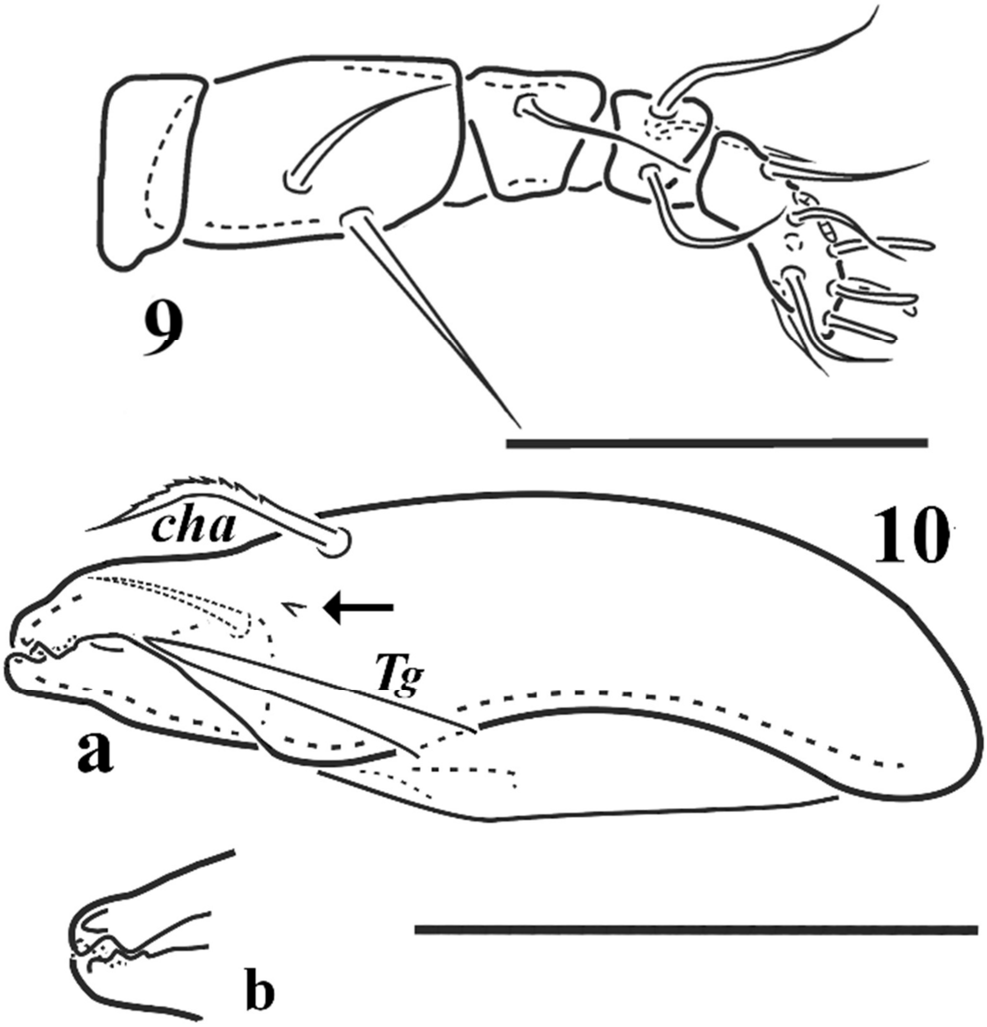

S2Figs. 9-10. *Caleremaeus mentobellus*: 9) right pedipalp abaxial; scale bar = 10 µm. 10) right chelicera, a) adaxial, b) distal end abaxial. *Tg* = Träghård's organ, arrow points to the spicule. Scale bar = 20 µm.

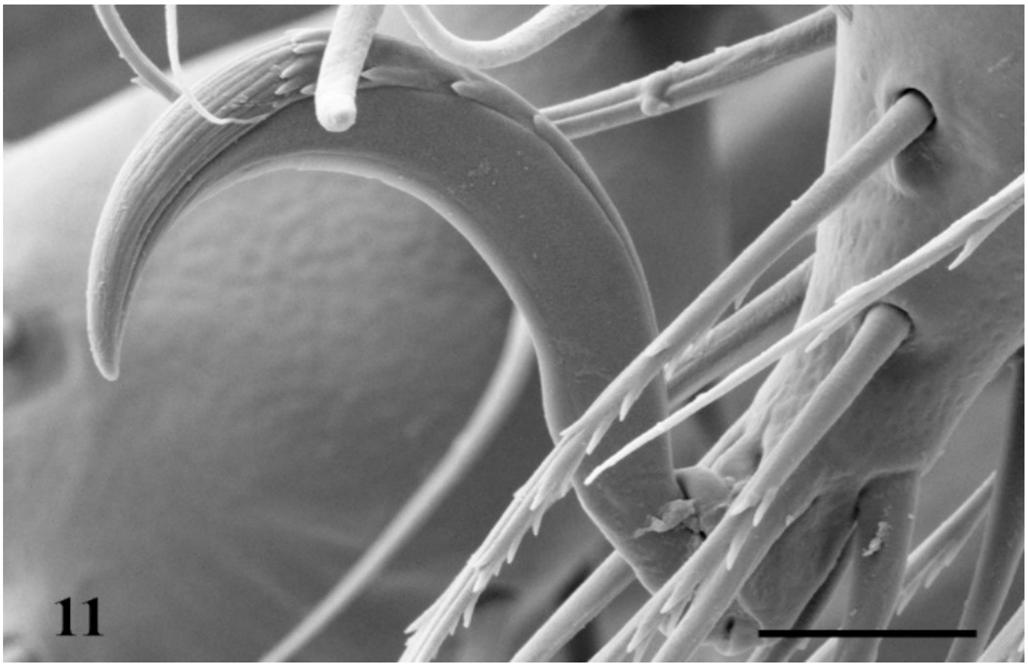

S2Fig. 11. *Caleremaeus alpinus*, claw of left leg I. SEM micrograph; scale bar = 5 µm.

Gnathosoma: Subcapitulum half-oval; diarthric (S2Fig. 7). Cuticle of genae and rutella smooth. Rutella separated from genae by a short incision. Rutellum stumpy, with one dorsal and one inner tooth; rutellar brush present. Lateral lips membranous, distal with two conical lappets instead of adoral setae (S2Fig. 43, *C. hispanicus*). Palpal setation (trochanter to tarsus, solenidion not included) 0-2-1-3-9 (S2Fig. 9); tarsal solenidion recumbent (S2Fig. 8). Chelicera (S2Fig. 10) chelate-dentate. Fixed digit with two terminal teeth and one subterminal tooth; moveable digit with two terminal teeth and three subterminal teeth. Träghård's organ (*T*) distinct, elongate and tapered. Setae *cha* and *chb* attenuate, *cha* barbed. Nearby seta *cha* one to three tiny cuticular spines, their number seems to be species-specific.

Ventral region: Narrow mentotectum (*mt*) and epimeral region with irregular arranged fovaeolae. Several enantiophyses and cuticular tubercles present across epimeral borders in varying numbers. The following enatiophyses are well developed in all studied specimens: enantiophyses e*Sa* and e*Sp* on lateral border of deep sejugal furrow as well as enantiophyses *e4a* and prominent cuspidate *e4p*; the latter pair spans deep epimeral groove IV laterally. Discidium (*dis*) directed laterally. Posterior border of epimeron IV with medial fossa; the contour of the fossa varies from trapezoid to parabolic. Epimeral setation 3-1-3-3; all setae smooth. Genital aperture slightly broader anteriorly. One pair of aggenital setae. Preanal organ club-shaped. Anal aperture broader posteriorly; each anal plate with two short setae. Three adanal setae, all smooth and similar in size. Lyrifissure *iad* paraanal.

Legs: All legs monodactylous, with big falcate claw consisting of two layers: an inner smooth one and an outer one with spines and striae (see S2Fig. 11). Femoral porose areas present as well as trochanteral porose areas on trochanters III and IV. On tarsus I famulus (ε) long with rounded tip. Genu I-III and tibia I-IV without seta *d*. Seta *tc´* on tarsus III very long and broadened. Solenidia: I (1-2-2), II (1-1-1), III (1-1-0), IV (0-1-0).

Caleremaeus monilipes (Michael, 1882)

Apart from Michael's description there is a generic diagnosis in Grandjean [3], and several short morphological notes for "*Caleremaeus monilipes*" are in determination keys; incomplete descriptions with figures are to be found in Subías & Arillo [4] and in Ayyildiz *et al.* [5]. Some figures are given by Miko & Travé [6]. Due to the different parts drawn and the varying quality of the figures given by these authors it is difficult to get an idea if these different morphological details belong to one and the same species. Seniczak & Seniczak [7] recently published a paper on the "Morphological ontogeny of *Caleremaeus monilipes* ..." based on specimens from Norway. Our morphological data are based on microscopic slides of Michael's collection from the Museum of Natural History of London. Slides labelled with: *Notaspis monilipes* Bred., 1930.8.25.707; *Notaspis monilipes* Parts, 1930.8.25.709.

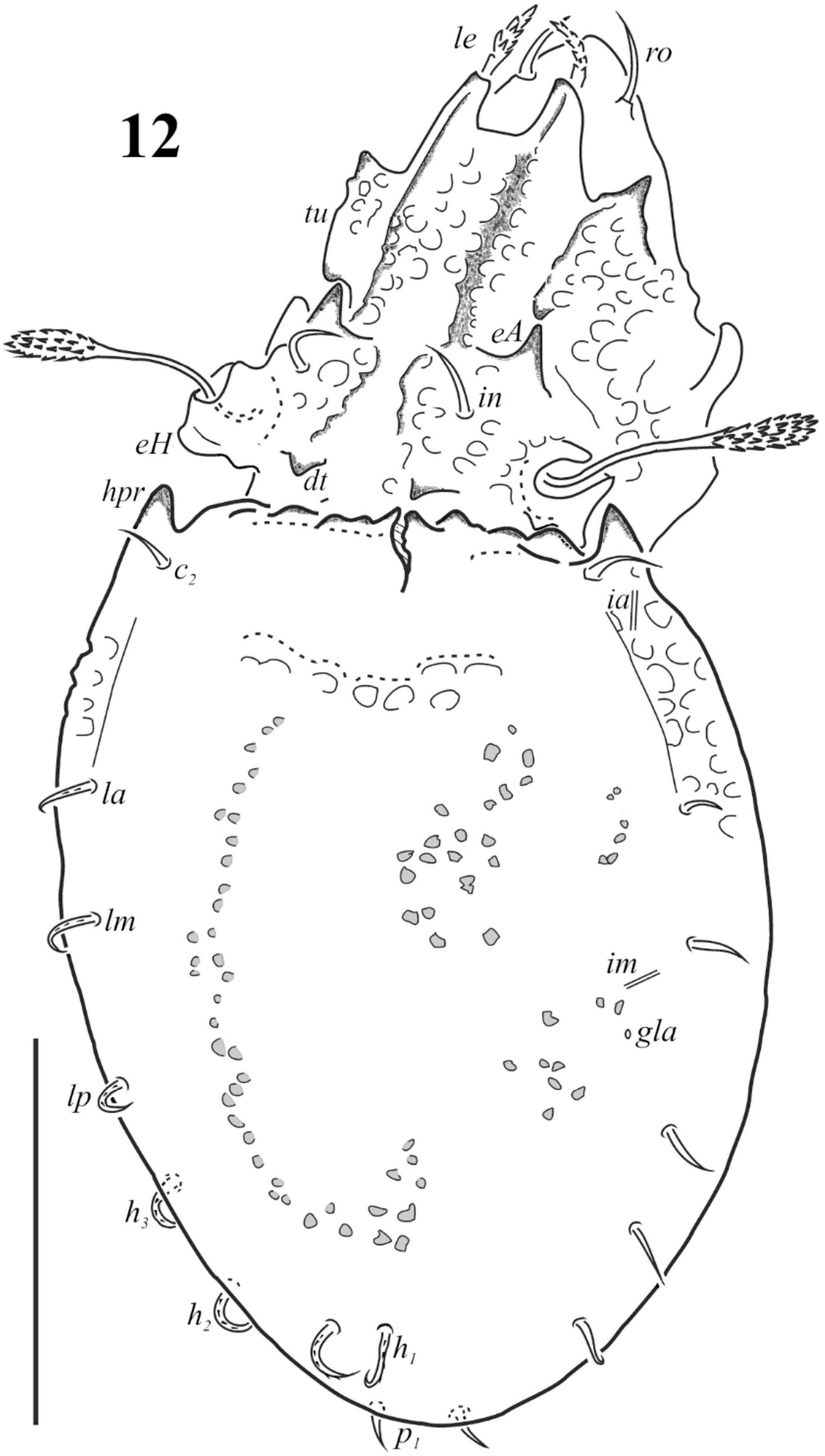

S2Fig. 12. *Caleremaeus monilipes*, dorsal view (microscopic slide of Michael's collection, 1930.8.25 707); cerotegument granules grey colored. Scale bar = 100 µm.

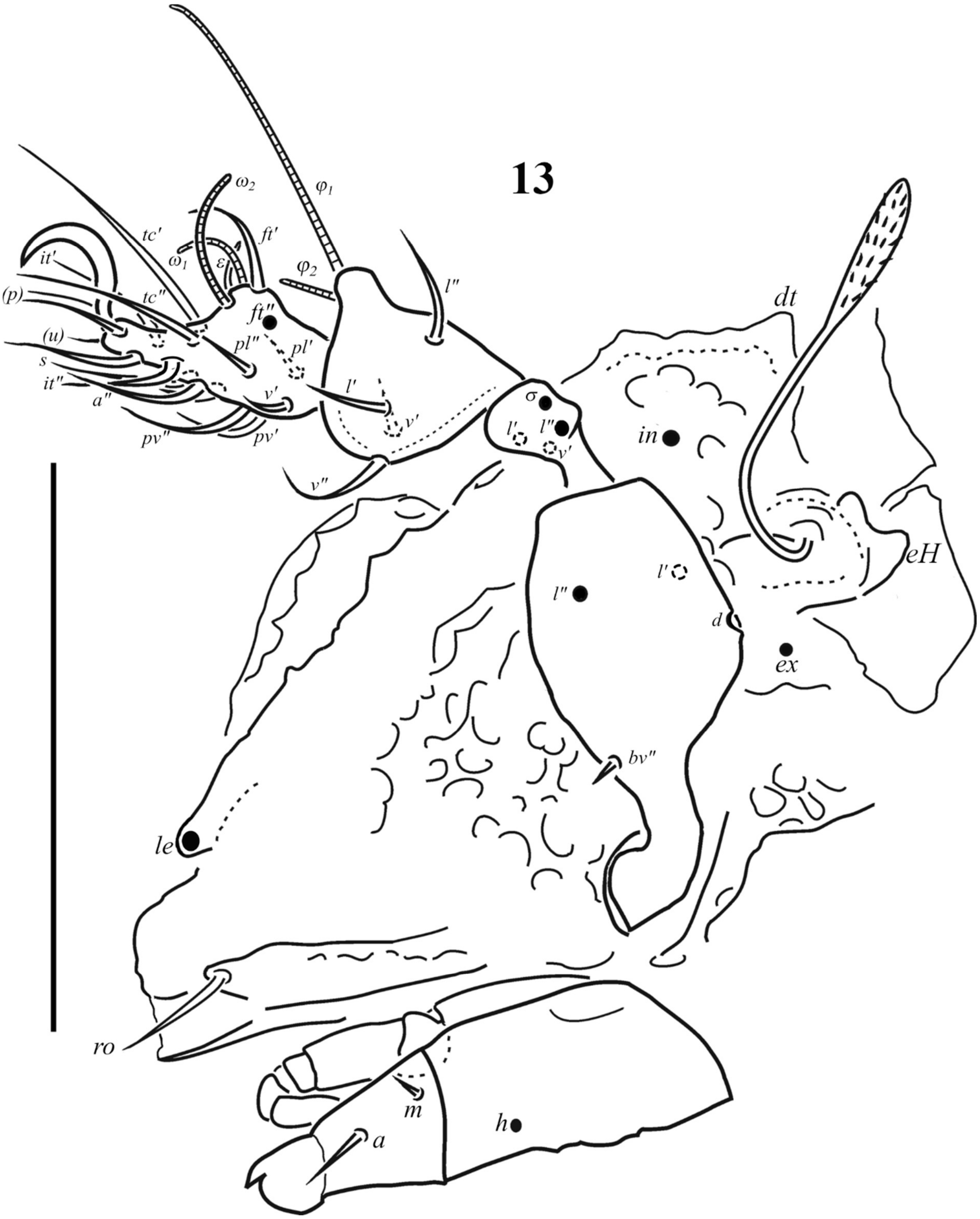

S2Fig. 13. *Caleremaeus monilipes*, lateral view; propodosoma, subcapitulum, and left leg I (microscopic slide of Michael's collection, 1930.8.25 709). Scale bar = 100 µm.

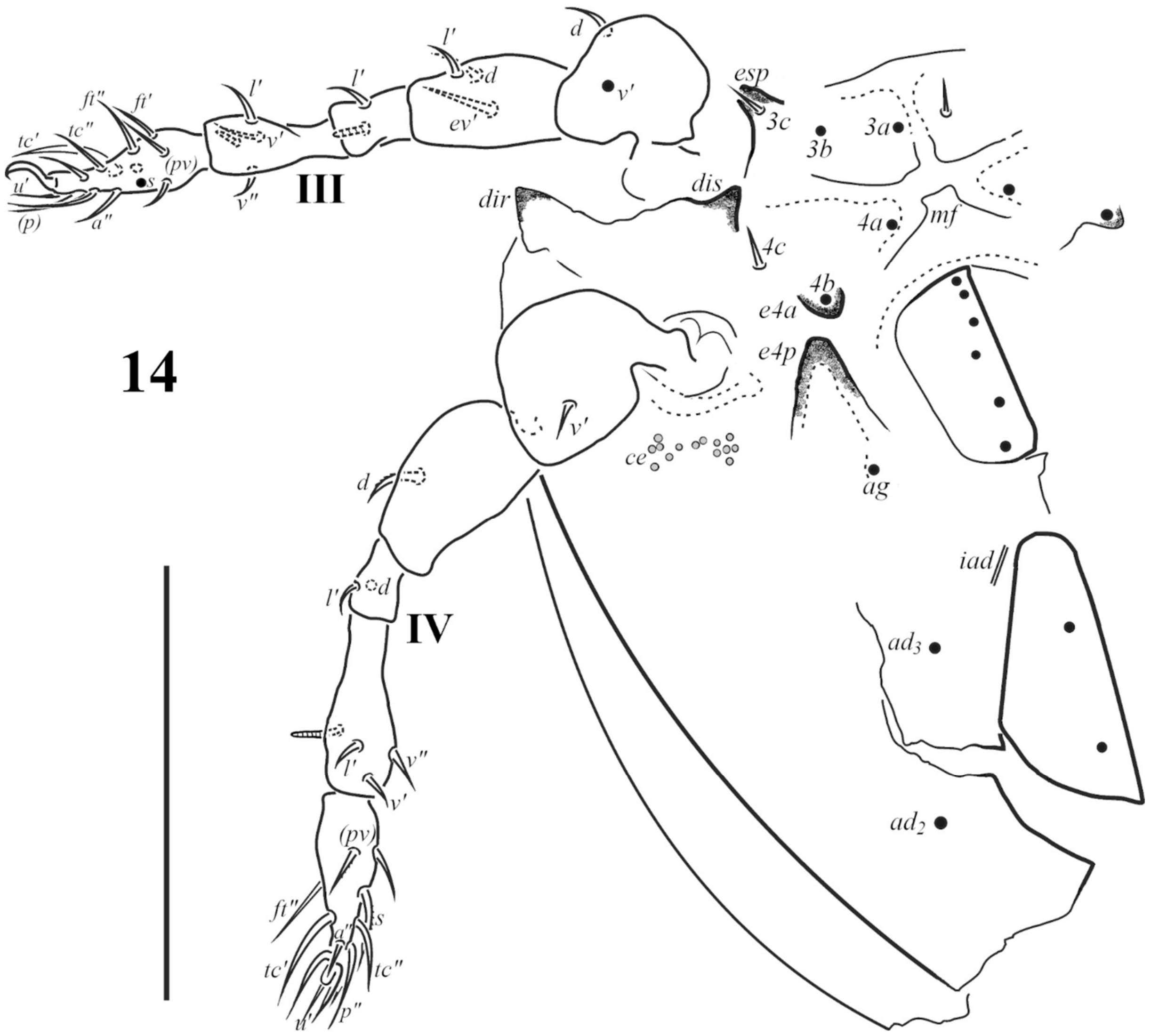

S2Fig. 14. *Caleremaeus monilipes*, ventral side; part of metapodo- and opisthosoma, leg III and IV (microscopic slide of Michael's collection, 1930.8.25 709), most of body setae broken. Scale bar = 100 µm.

Diagnosis: Mentum without foveae, its cuticular surface finely granulate. Seta *d* and *l'* very stout and spinose on femur I and II. Dorsal seta of femur III broad and serrate. Femur IV only with one seta.

Species characters: Length: 363 µm, width 190 µm; (in Michael [2]: 340/180 µm).

Dorsal view see S2Fig. 12, lateral aspect of propodosoma and subcapitulum S2Fig. 13.

Prodorsum: Basal part of bothridium with two enantiophyses directed to notogaster, the adaxial one very small. Bothridial seta spinose, long, with slender club.

Cerotegument forming irregular nodules on notogaster. Notogastral setae robust, h_1 very close to each other.

Mentum without foveae, its cuticular surface finely granulate. On adaxial side of chelicera two small cuticular spines below insertion of seta *cha*.

Ventral side (S2Fig. 14) with well-developed discidium and enantiophyses *eSa*, *eSp*, and, *e4a* and *e4p*. Six pairs of genital setae.

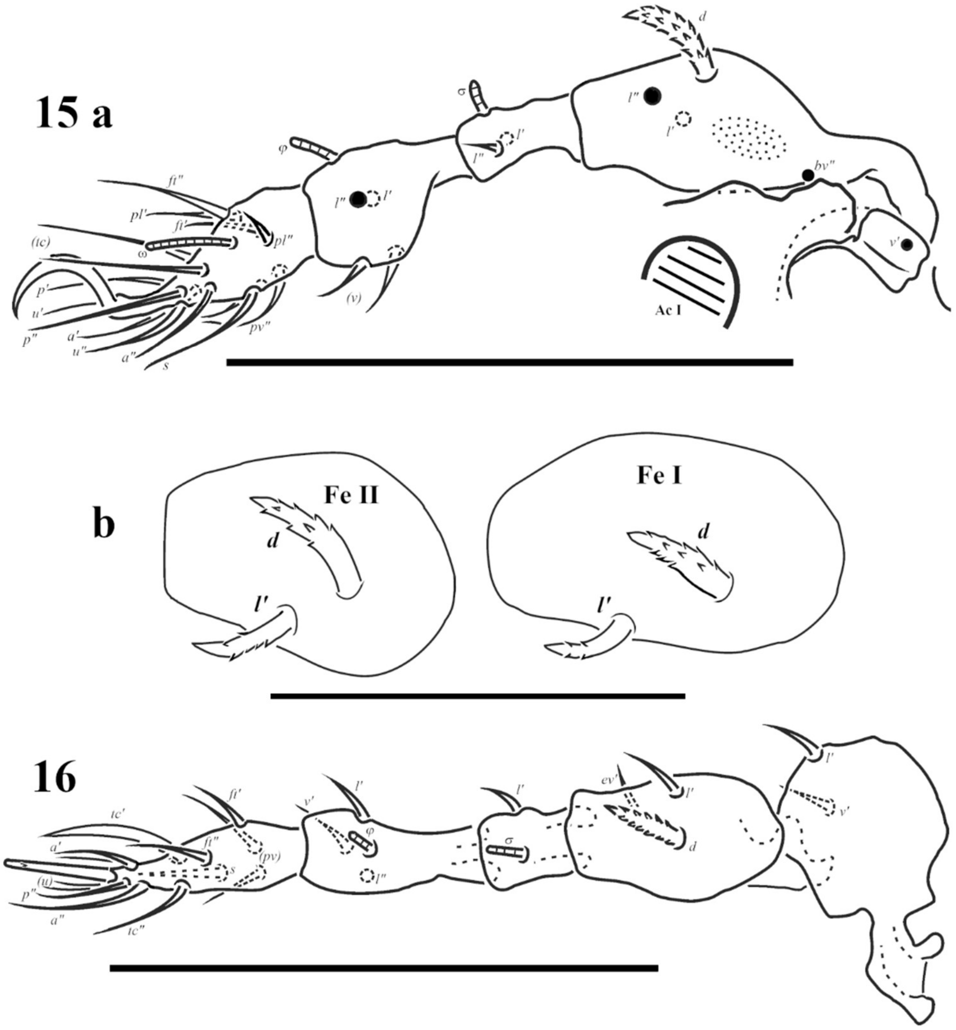

S2Figs. 15-16. *Caleremaeus monilipes*: 15) a) left leg II, abaxial, scale bar = 100 µm; b) seta d + l′ of femur I and II, only dorsal parts of femora drawn (microscopic slide of Michael's collection, 1930.8.25 709). Scale bar = 50 µm. 16) left leg III, dorsal view; dorsal seta of femur broad and serrate. Scale bar = 100 µm.

Setal formulae: leg I (1-4-3-4-19), seta *l″* on tarsus missing (S2Fig. 13); leg II (1-4-2-4-15), seta *it′* on tarsus missing (S2Fig. 15a); leg III (2-3-1-3-13), seta *it″* on tarsus missing (S2Fig. 14); leg IV (1-1-2-3-11), seta *ev′* on femur missing (S2Fig.14). Seta *d* and *l″* very stout and spinose on femur I and II (S2Fig. 15b). Dorsal seta of femur III broad and serrate (S2Fig. 16). Claws slender.

Caleremaeus mentobellus sp. nov.

Species Diagnosis: Average length 360 µm, average body width 187 µm. Bothridial seta clavate and spinose. 10 pairs of spiniform, slightly barbate notogastral setae; setae h_1 longest. Anterior half of mentum with three transverse rows of foveae becoming smaller posteriorly. On femur I and II seta *d* stout and spinose, seta *l′* spiniform and smooth.

Body size and appearance: Holotype length 371µm, width 215 µm. Mean total length 360 µm (n 23, range 333 – 386 µm); mean notogastral width 187 µm (n 23, range 170 – 215 µm). Dorsal aspect see S2Fig. 17. Colour light to medium brown. Cerotegument on notogaster forming globular nodules (diameter about 2 µm) (S2Fig. 19a), similar in epimeral and anogenital region but in protected regions of the lateral podosoma (see S2Fig. 3) with micro-tuberculate basal layer bearing small granules composed of peg-shaped elements (diameter about 1 µm) (S2Fig. 19b). Micro-tubercles evenly distributed.

Prodorsum (S2Figs. 3, 17): Cuticle with numerous rounded foveae dorsally and laterally, except central proximal part. Basal part of posterior wall of bothridium with two rounded platelets directed to border of notogaster, the exterior one larger forming the enantiophyses *eH*. Bothridial seta clavate, spinose. Rostrum slightly contoured because of irregular cuticular ledges and pits. Rostral setae (*ro*) similar in size as *in*.

Ventral region (S2Fig. 18): Several enantiophyses present across epimeral borders except epimeral border I. Genital plates each with six smooth setae; distance between *g5* and *g6* longer than between other setae.

Gnathosoma: Mentum in anterior half with approximately three rows of foveae; in the central area cuticle with fine wrinkles. Anterior border of mentotectum medially slightly bulged. Setae *h* short and smooth. Genal setae (*a, m*) long, *m* delicately pectinate (S2Fig. 20). Pedipalp (S2Figs. 8, 9) and chelicera (S2Fig. 10); on chelicera one to two small cuticular spines below insertion of seta *cha*.

Legs: Leg I to IV (S2Fig. 21). Setal formulae: leg I (1-4-3-4-20); leg II (1-4-2-4-16) leg III (2-3-1-3-14); leg IV (1-2-2-3-11). [In one case we found on legs I to III the tarsal setae reduced to 19, 15, 13 respectively]. On femur I and II seta *d* stout and spinose, seta *l″* spiniform and smooth on femur I, on femur II with tiny barbs (S2Fig. 22).

Derivatio nominis: "*mentobellus*" means a beautiful mentum, ornamented with many foveae in the anterior half of mentum.

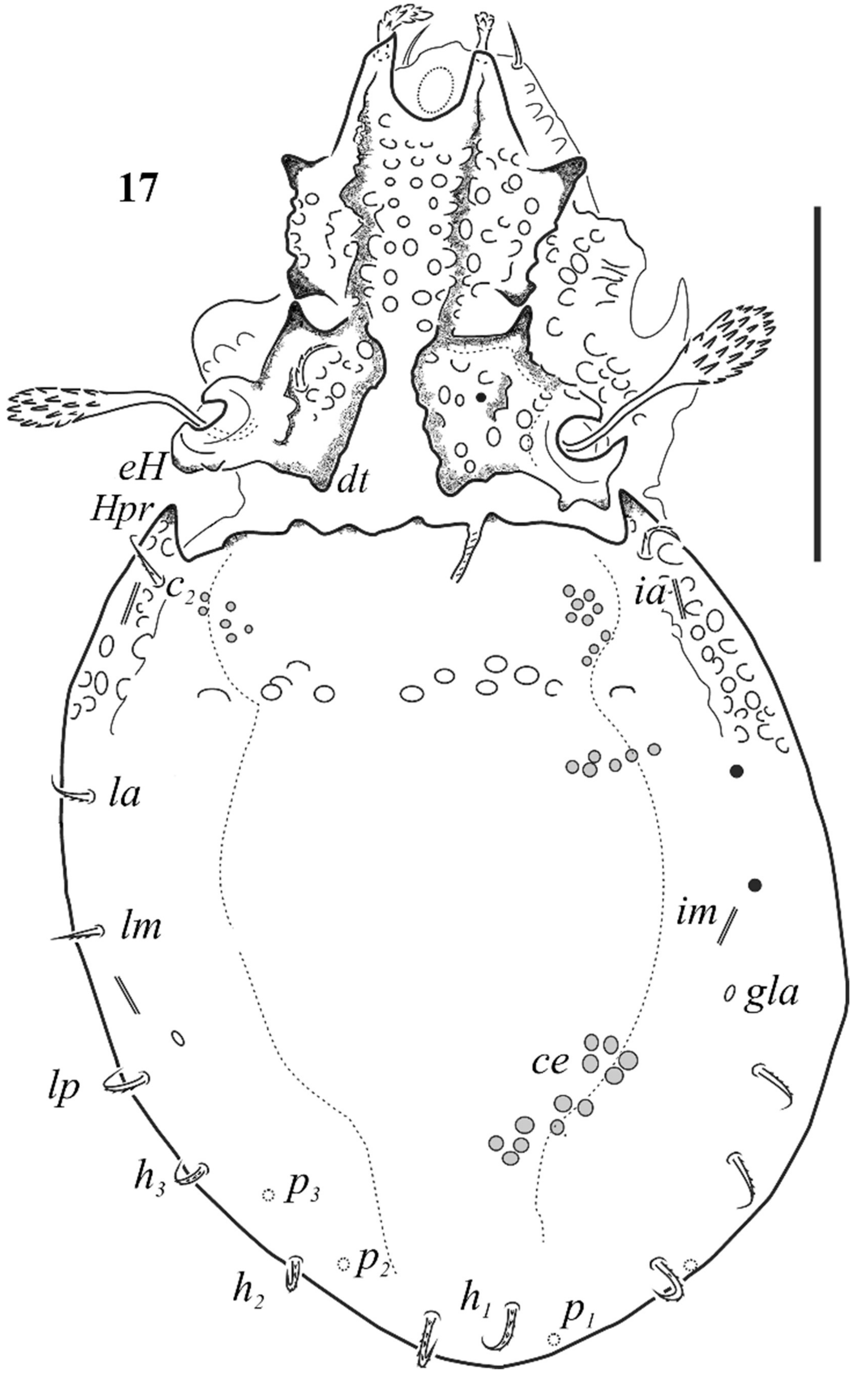

S2FigS. 17. *Caleremaeus mentobellus*: dorsal view (anterior border of notogaster broken). Scale bar = 100 µm.

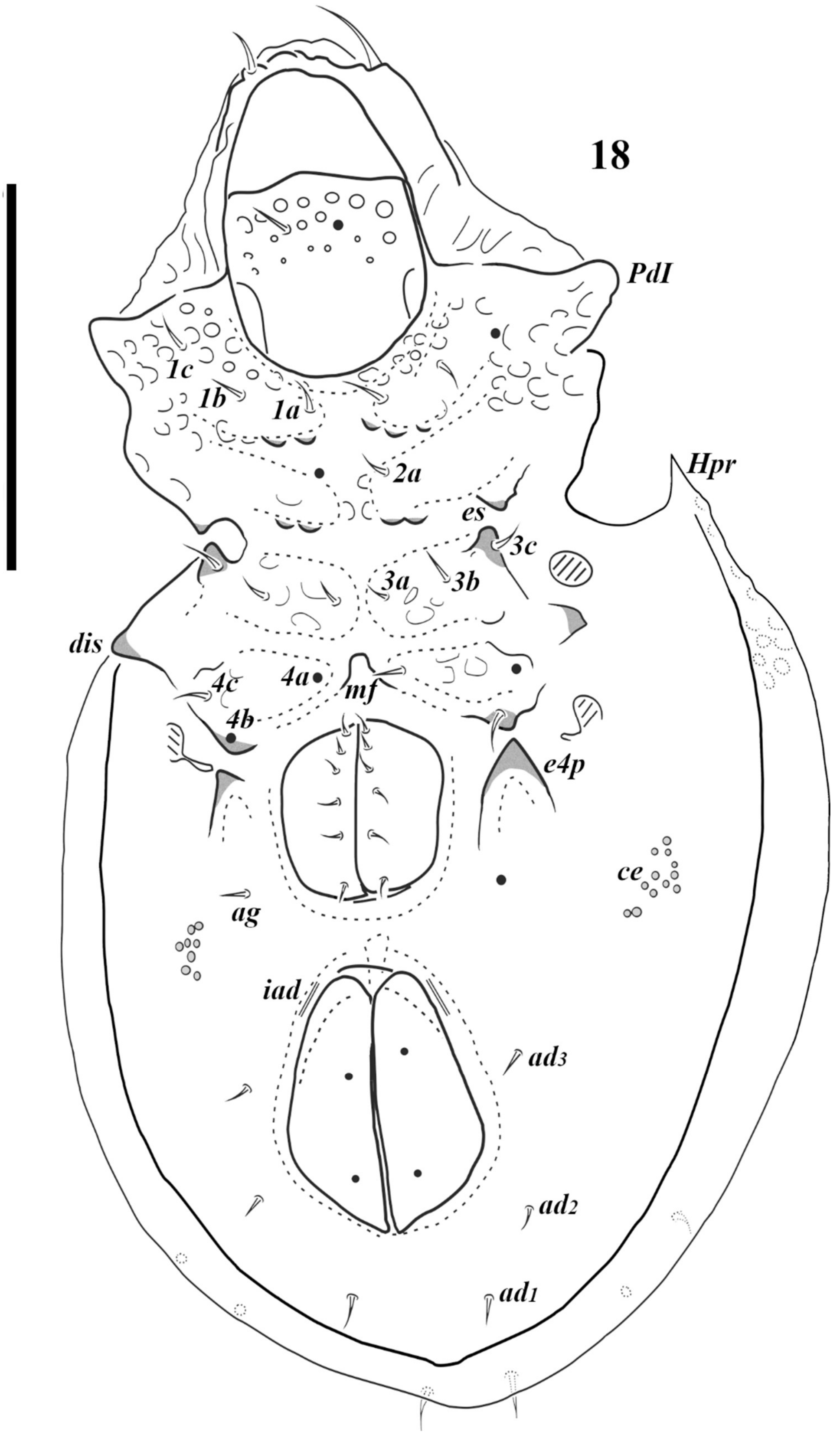

S2FigS. 18. *Caleremaeus mentobellus*: ventral view. Scale bar = 100 µm.

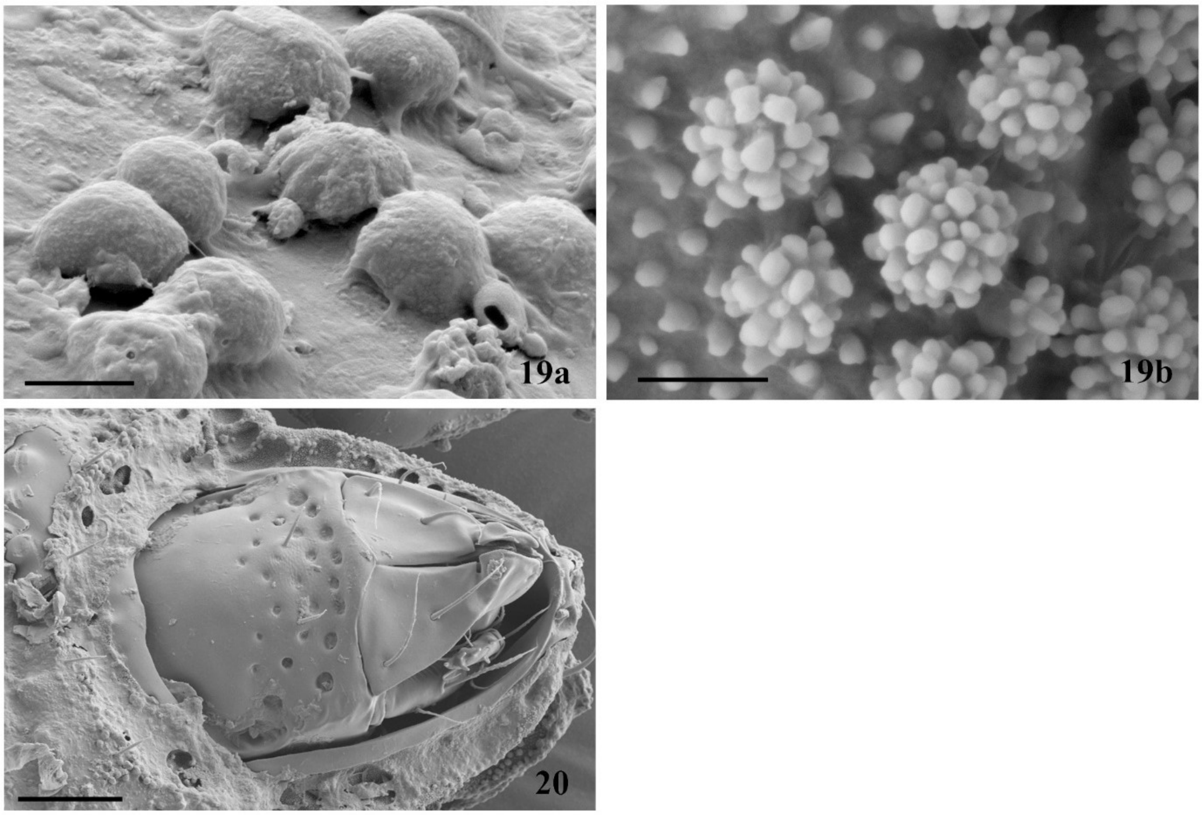

S2Figs. 19-20. *Caleremaeus mentobellus*: SEM micrographs. 19 a) cerotegument nodules on notogaster, Scale bar = 2 µm; b) podosoma lateral; fine structure of cerotegumental grains and microtubercles. Scale bar = 1 µm. 20) ventral view, subcapitulum; mentum with approx. three rows of foveae, cuticle in central part with fine wrinkles. Scale bar = 20 µm.

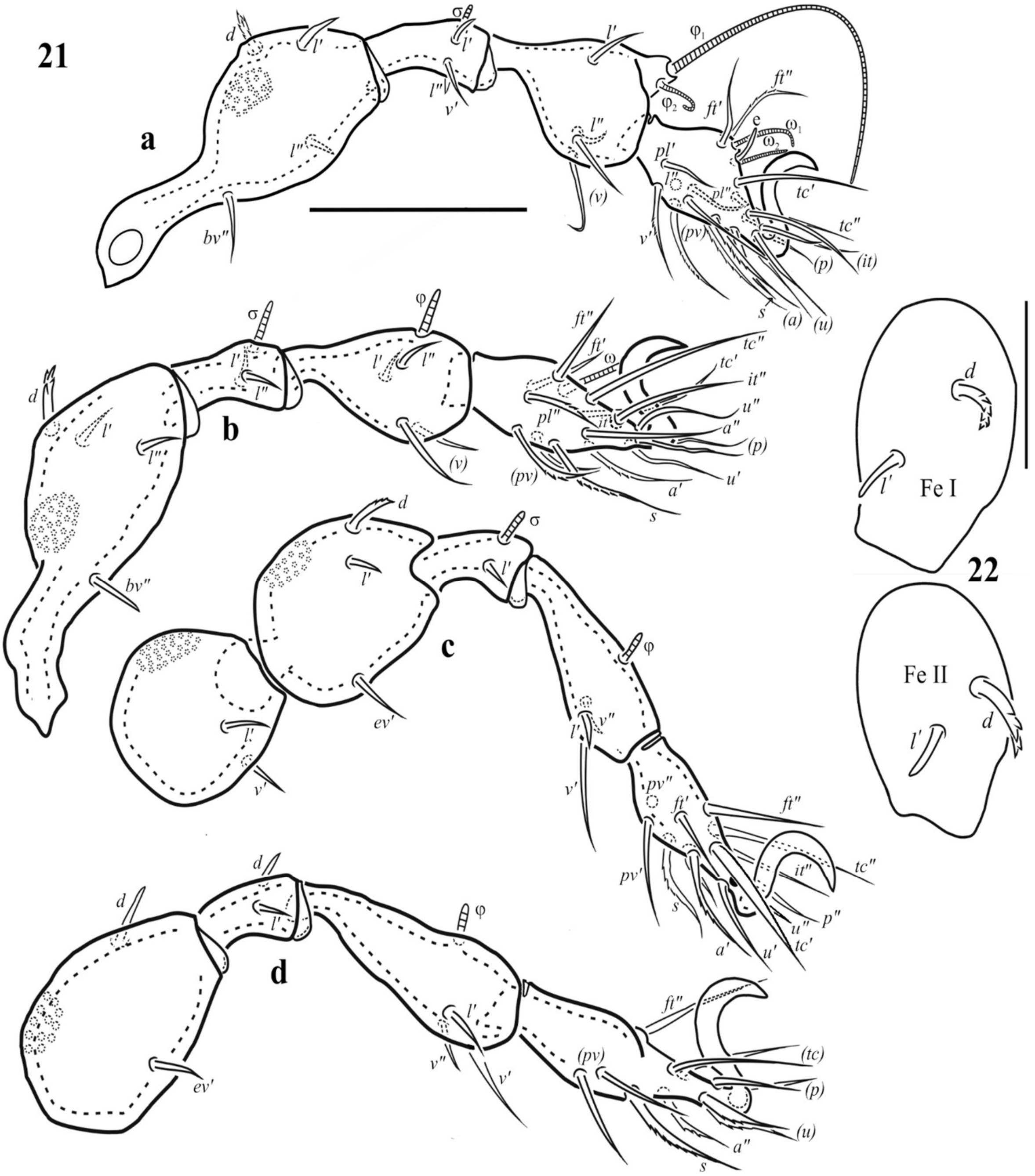

S2Figs. 21-22. *Caleremaeus mentobellus*: 21) legs I-IV: a) left leg I adaxial, b) right leg II abaxial, c) left leg III abaxial, left leg IV abaxial (trochanter not drawn). Scale bar = 50 µm. 22) left femur I and II in dorsal aspect. Scale bar = 25 µm.

Caleremaeus lignophilus sp. nov.

Species Diagnosis: Average length 333 µm, average body width 168 µm. Mentum on anterior border with one row of shallow foveae; surface of cuticle finely granulated. Border of mentotectum medially only slightly projecting. Setae *d* on femur I and II stout and spinose, setae *l'* spiniform and with tiny barbs.

Body size and appearance: Holotype length 361 µm, width 189 µm. Mean total length 333 µm (n 41, range 307 – 367 µm); mean notogastral width 168 µm (range 156 – 194 µm). Colour light to medium brown. Cerotegument on notogaster forming comparatively big mushroom-like nodules with irregular outline (diameter 3 – 7 µm) (S2Fig. 24), in epimeral and anogenital region smaller nodules. Fine structure of cerotegument in lateral region of podosoma the same as in *C. mentobellus*.

Prodorsum, **notogaster**, and **ventral region** very similar to *C. mentobellus* (S2Figs. 23, 25, 27, 28).

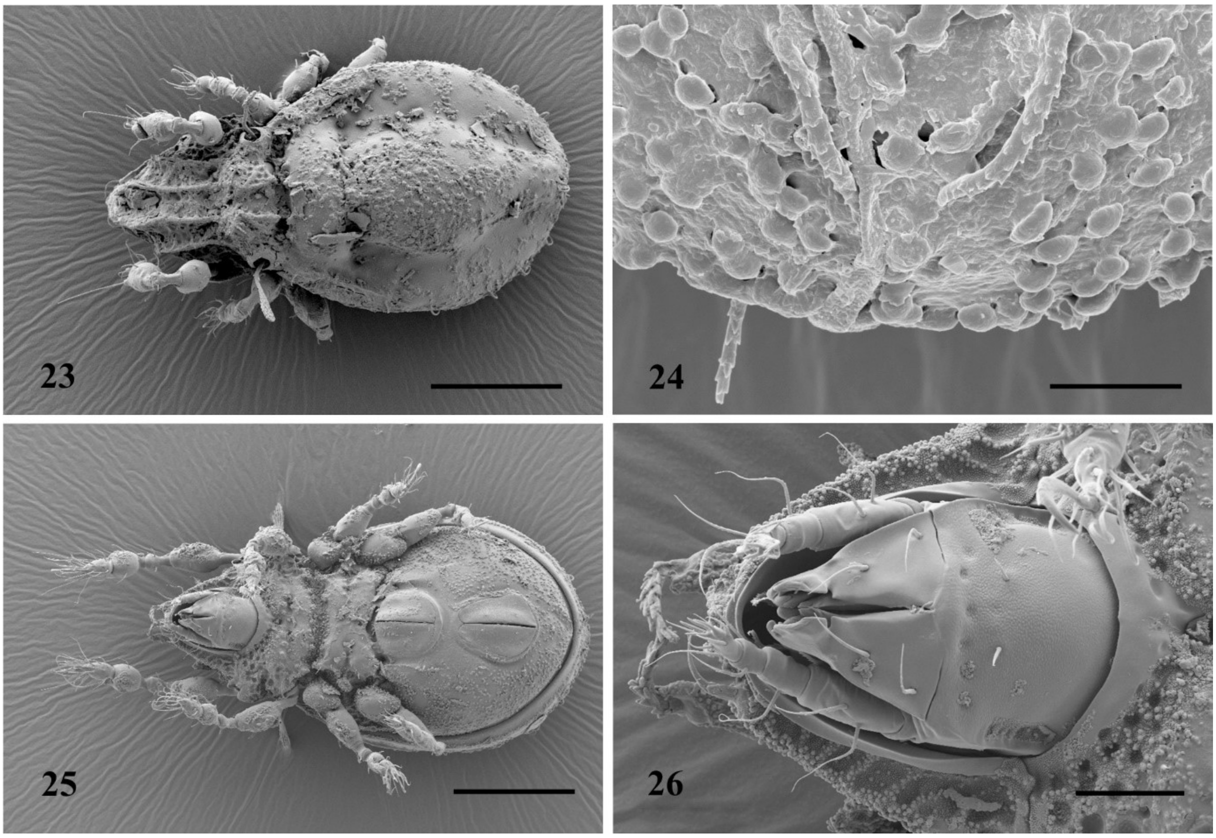

S2Figs. 23-26. *Caleremaeus lignophilus*: SEM micrographs. 23) dorsal view; notogastral cerotegument partly detaching. Scale bar = 100 µm. 24) posterior part of notogaster; mushroom-like cerotegument nodules. Scale bar = 10 µm. 25) ventral view. Scale bar = 100 µm. 26) ventral view, subcapitulum and pedipalps; mentum with one row of foveae, cuticle finely granulated. Scale bar = 20 µm.

Gnathosoma: Mentum on anterior border with one row of shallow foveae; surface of cuticle finely granulated. Setae *h* short and smooth. Genal setae (*a, m*) long, *m* delicately barbed (S2Fig. 26). Border of mentotectum medially only slightly projecting.

Chelicera: Close to seta *cha* three tiny cuticular spines.

Legs: Setal formulae: leg I (1-4-3-4-20); leg II (1-4-2-4-16) leg III (2-3-1-3-14); leg IV (1-2-2-3-11). Seta *d* on femur I and II stout and spinose, seta *l'* spiniform and with tiny barbs (S2Fig. 29). Femur IV with two setae.

Derivatio nominis: "*lignophilus*" means that the animals like decaying wood; this species was found exclusively in samples of decaying wood.

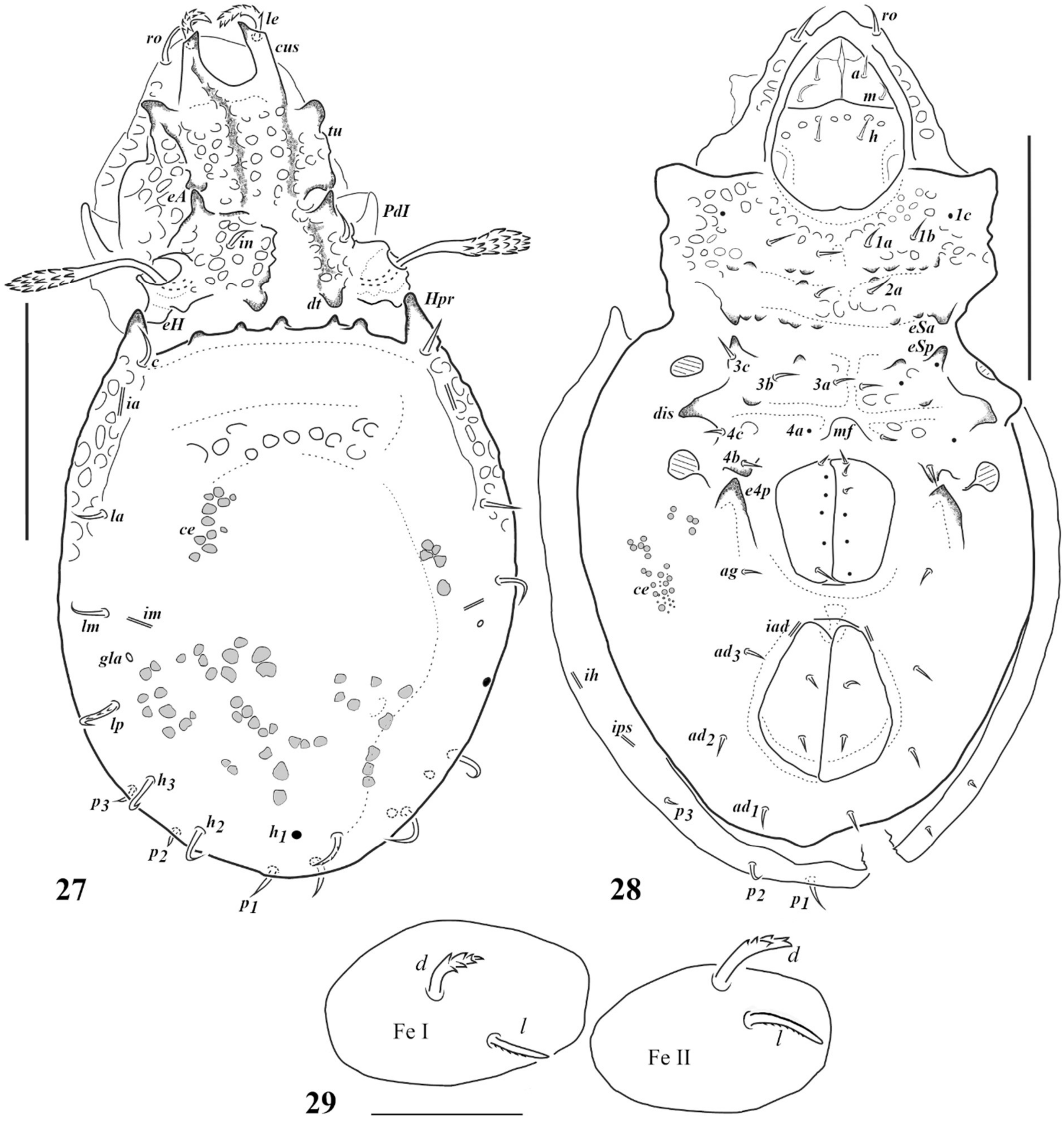

S2Figs. 27-29. *Caleremaeus lignophilus*: 27) dorsal view. Scale bar = 100 µm. 28) ventral view. Scale bar = 100 µm. 29) left femur I and II in dorsal aspect (only dorsal parts of femora drawn). Scale bar = 25 µm.

Caleremaeus elevatus sp. nov.

Species Diagnosis: Average length 353 µm, average body width 194 µm. Foveae on anterior half of mentum arranged irregularely. Five pairs of genital setae. Seta *d* as well as seta *l′* on femur I and II spiniform and smooth.

Body size: Holotype length 342 µm, width 201 µm. Mean total length 353 µm (n 16, range 322 – 376 µm); mean notogastral width 194 µm (range 163 – 220 µm).

Prodorsum: Surface less foveate than in the other species. Basal part of posterior wall of bothridium with only one rounded platelet directed to border of notogaster − enantiophysis *eH* (S2Fig. 30). **Ventral side** (S2Fig. 31): Only few enantiophyses along epimeral borders, mainly on posterior border of epimeron I. Enantiophyses *e4p* well developed. Discidium developed weakly. Five pairs of genital setae. In one case anal setae 1+2 instead of 2+2.

Gnathosoma: Numerous foveae on anterior half of mentum arranged irregularely. Border of mentotectum without median projection.

Chelicera: Below insertion of seta *cha* three very small cuticular spines.

Legs: Setal formulae: leg I (1-4-3-4-20); leg II (1-4-2-4-16) leg III (2-3-1-3-14); leg IV (1-2-2-3-11). Seta *d* as well as seta *l′* on femur I and II spiniform and smooth (S2Fig. 32). Femur IV with two setae.

Derivatio nominis: "*elevatus*" means to ascend elevated spots because always found on sun exposed rocks or roofs above the soil.

Caleremaeus alpinus sp. nov.

Species Diagnosis: Average length 379 µm, average body width 197 µm. Few foveae on anterior half of mentum arranged irregularely; cuticle in the area of foveae rugose. On femur I and II seta *d* stout and spinose, seta *l′* spiniform and smooth on femur I, on femur II with tiny barbs.

Body size: Holotype length 417 µm, width 222 µm. Mean total length 379 µm (n 39, range 353 – 417 µm); mean notogastral width 197 µm (range 177 – 222 µm).

Dorsal side see S2Fig. 33. **Prodorsum**: Basal part of posterior wall of bothridium with two rounded platelets directed to border of notogaster. Interlamellar setae relatively tenuous.

Ventral side (S2Fig. 34): Several enantiophyses along posterior borders of epimeron I and II. Six pairs of genital setae. Cerotegumental microtubercels on border of ventral plate evenly distributed.

Gnathosoma: Few foveae on anterior half of mentum arranged irregularly; border of foveae not well discernible in transmitted light microscopy. Cuticle in the area of foveae finely rugose. Border of mentotectum without median projection (S2Fig. 36). **Chelicera**: Below insertion of seta *cha* three very small cuticular spines.

Legs: Setal formulae: leg I (1-4-3-4-20); leg II (1-4-2-4-16) leg III (2-3-1-3-14); leg IV (1-2-2-3-11). On femur I and II seta *d* stout and spinose, seta *l′* spiniform and smooth on femur I, on femur II with tiny barbs (S2Fig. 35). Femur IV with two setae.

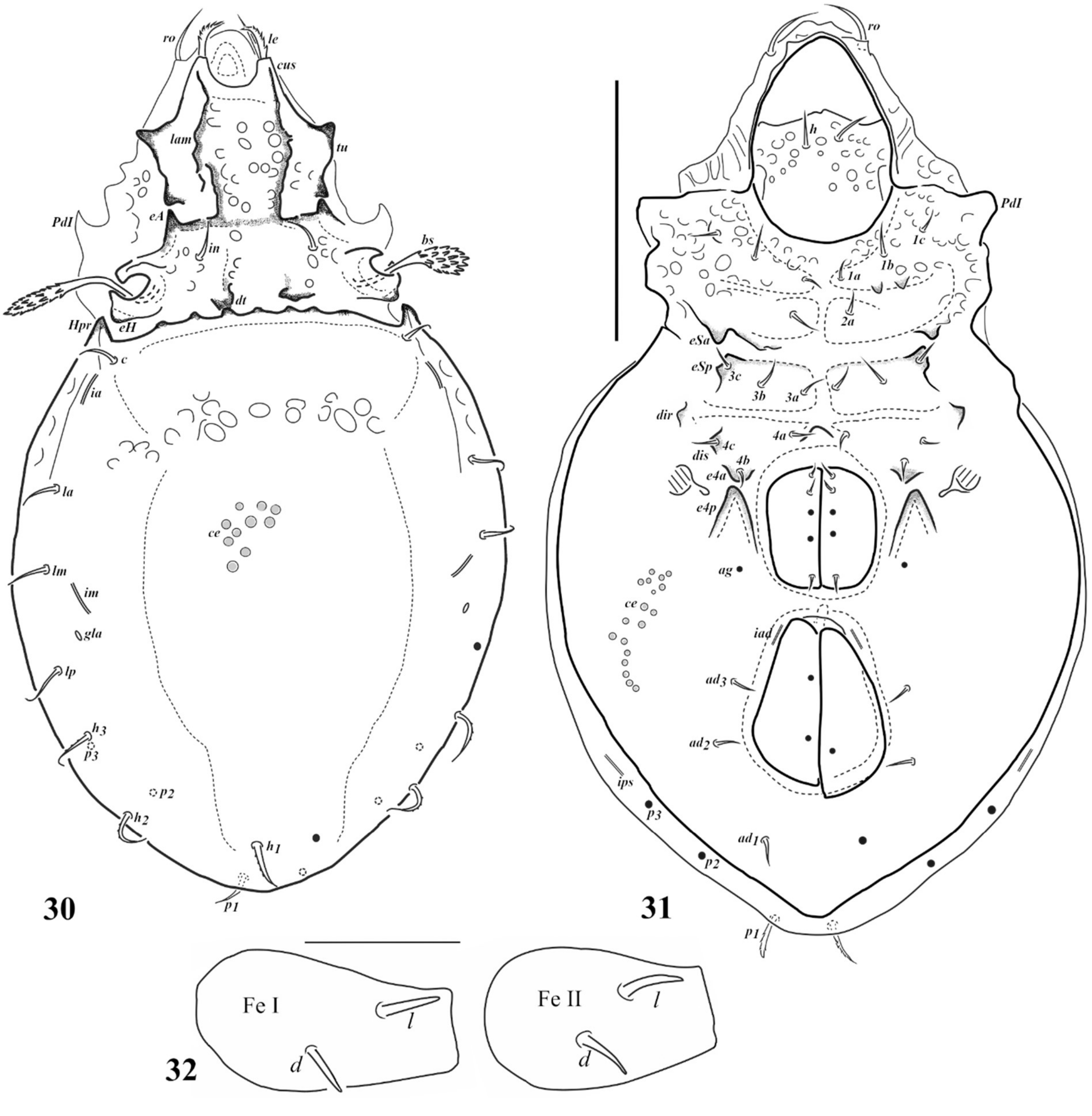

S2Figs. 30-32. *Caleremaeus elevatus*: 30) dorsal view. Scale bar =100 µm. 31) ventral view. Scale bar =100 µm. 32) right femur I and II in dorsal aspect (only dorsal parts of femora drawn). Scale bar = 25 µm.

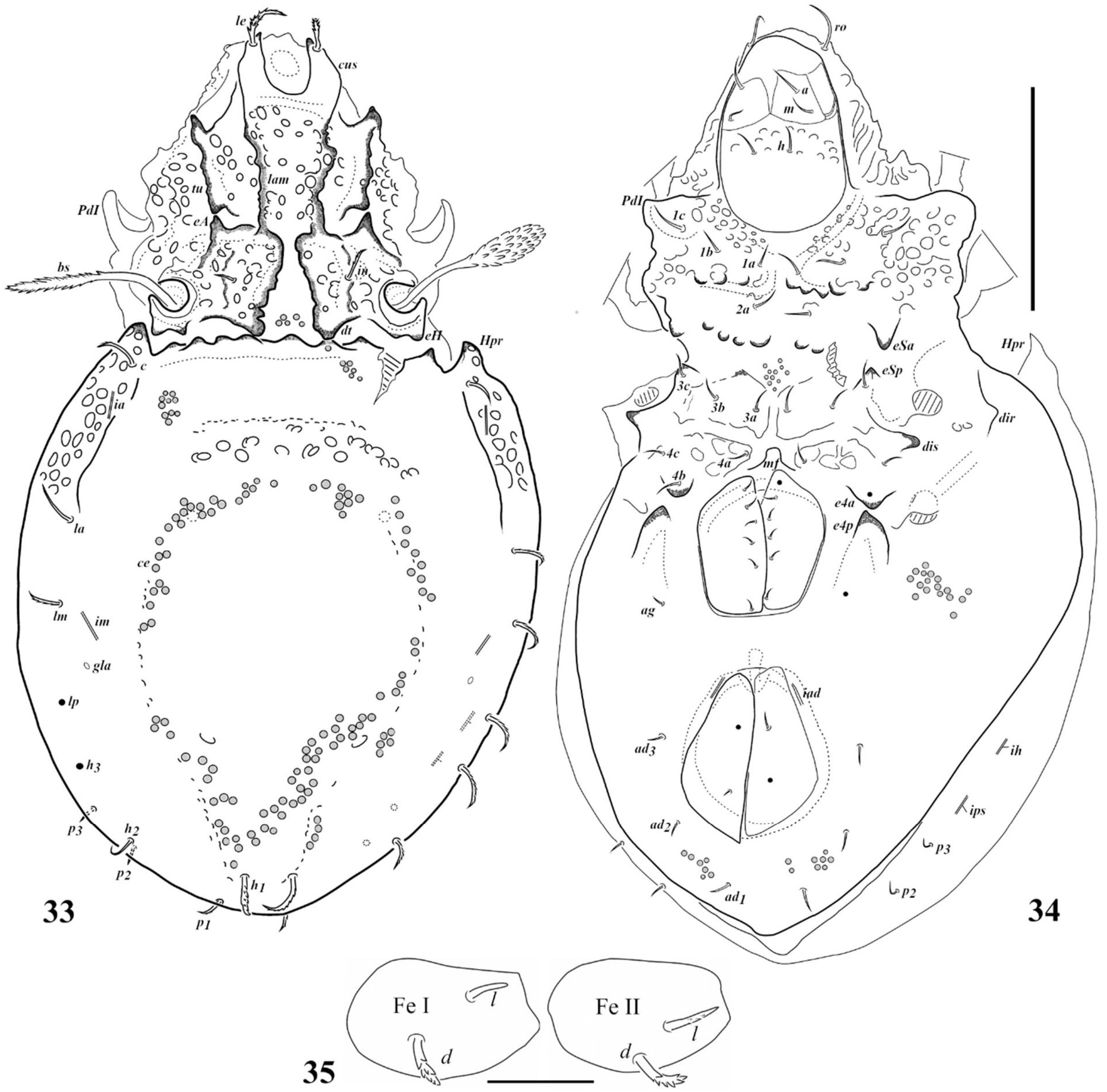

S2Figs. 33-35. *Caleremaeus alpinus*: 33) dorsal view; anterior border of notogaster broken. Scale bar =100 µm. 34) ventral view. Scale bar =100 µm. 35) right femur I and II in dorsal aspect (only dorsal parts of femora drawn). Scale bar = 25 µm.

Derivatio nominis: specimens of this species occur only at higher altitudes, from the subalpine to the alpine zone, and therefore named as “*alpinus*”.

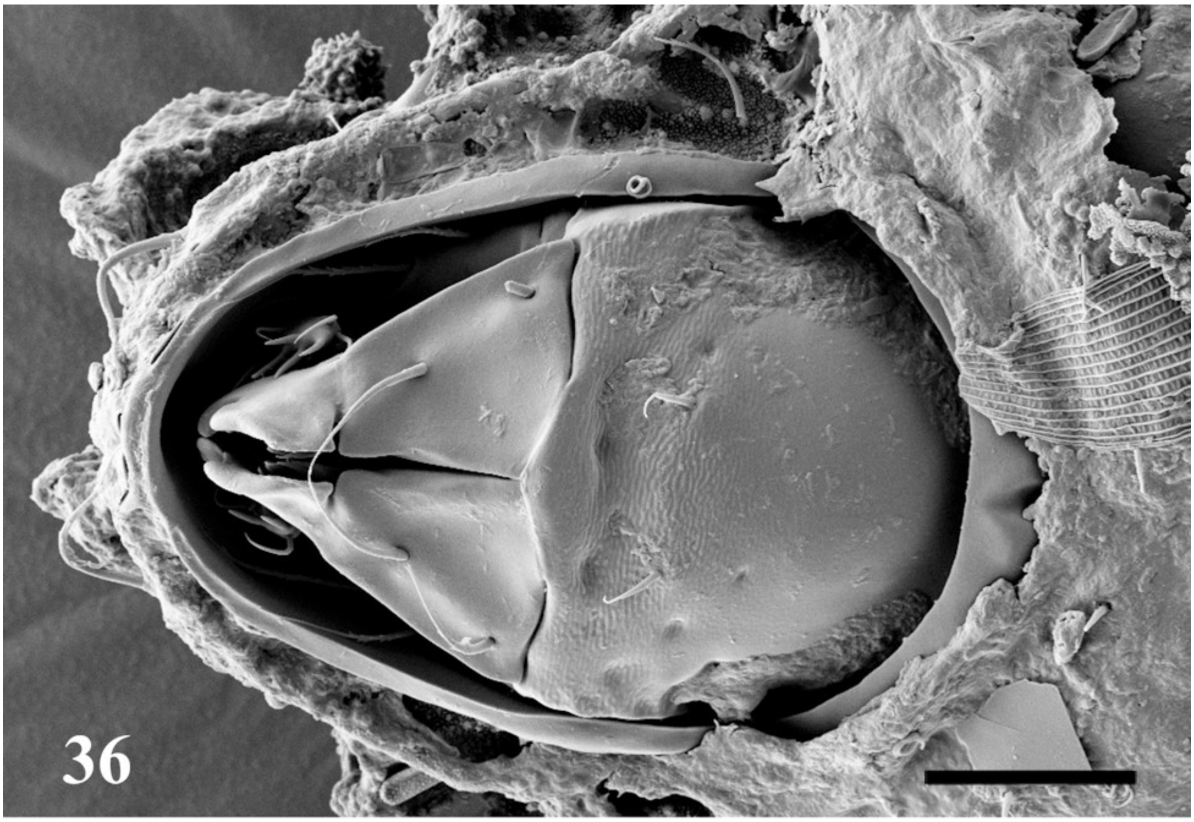

S2Fig. 36. *Caleremaeus alpinus*: SEM micrograph. Subcapitulum, ventral view; mentum with few irregularly arranged foveae, cuticle finely rugose. Scale bar = 20 µm.

Caleremaeus hispanicus sp. nov.

Species Diagnosis: Average length 370 µm, average body width 196 µm. Mentum without foveae, cuticle of distal half finely wrinkled. On femur I seta *d* small and spinose, seta *l′* fine and smooth. On femur II seta *d* and *l′* stout and spinose.

Body size and appearance: Holotype length 374 µm, width 199 µm. Mean total length 370 µm (n 6, range 362 – 379 µm); mean notogastral width 196 µm (range 192 – 204 µm). Habitus dorsal see S2Fig. 37.

Prodorsum: Basal part of posterior wall of bothridium with only one rounded platelet directed to border of notogaster - enantiophysis e*H*. Rostral setae a bit more slender than in *C. mentobellus*.

Notogaster: Mushroom-like, relatively large notogastral cerotegument grains (diameter about 4 µm) with irregular border (S2Fig. 40 a, b). Notogastral setae relatively long, h_1 longest and close to each other.

Ventral side (S2Figs. 38, 41): Epimeral region only anterior with many foveae, in the middle and posterior part foveae only weakly developed. Few enantiophyses along posterior borders of epimeron I and II. Discidium small. Six pairs of genital setae. Microtubercles of cerotegument arranged in rows on lateral border of ventral plate (S2Fig. 42).
Mentum without foveae, cuticle of anterior half finely rugose (S2Fig. 43); lateral lips S2Fig. 44.
Legs: Setal formulae: leg I (1-4-3-4-20); leg II (1-4-2-4-16) leg III (2-3-1-3-14); leg IV (1-2-2-3-11). On femur I seta *d* small and spinose, seta *l′* fine and smooth. On femur II seta *d* and *l′* stout and spinose (S2Fig. 39)
Derivatio nominis: “hispanicus” refers to Spain from where the species was collected.

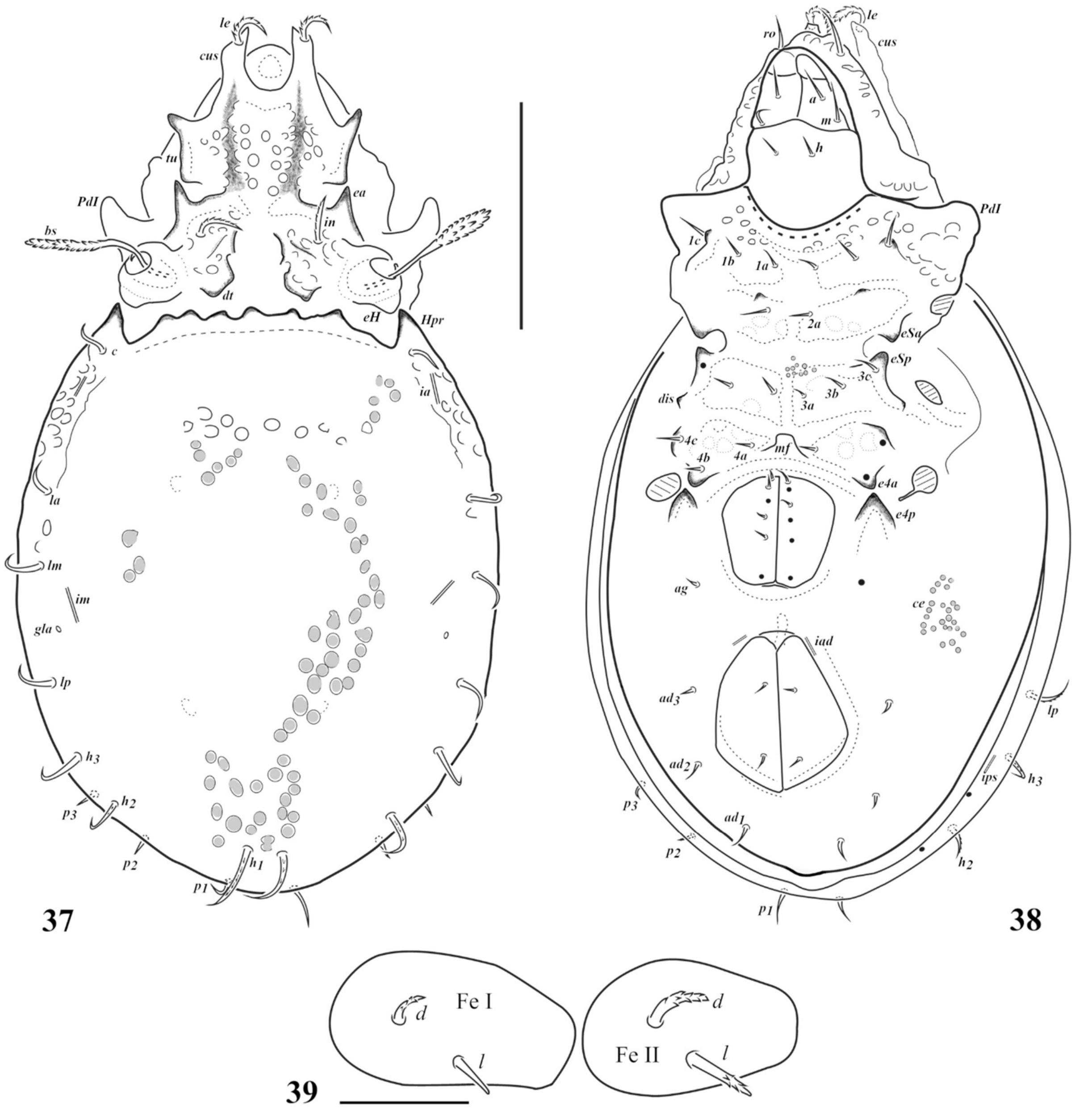

S2Figs. 37-39. *Caleremaeus hispanicus*: 37) dorsal view. Scale bar =100 µm. 38) ventral view. Scale bar =100 µm. 39) left femur I and II in dorsal aspect (only dorsal parts of femora drawn). Scale bar = 25 µm.

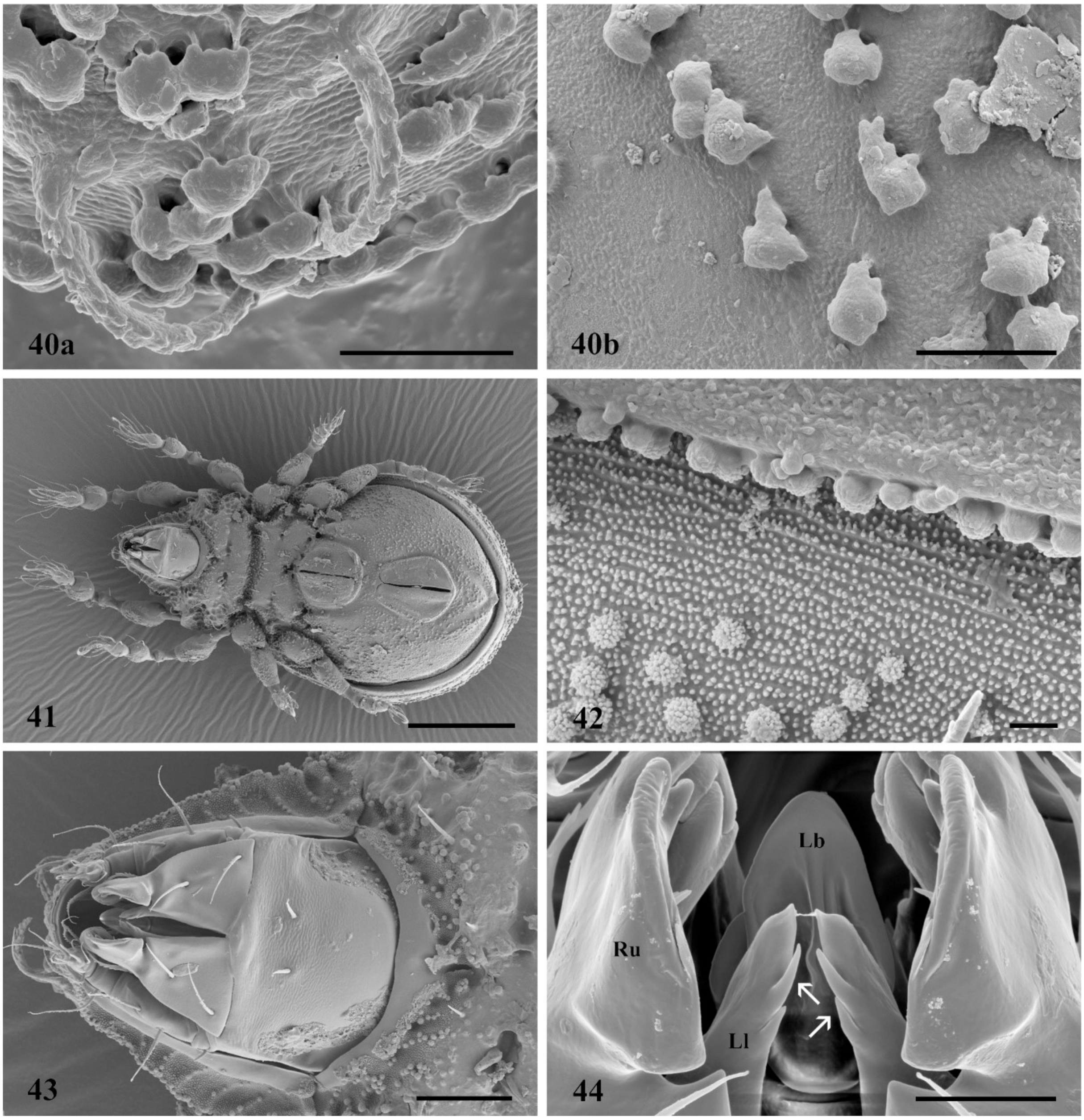

S2Figs. 40-44. *Caleremaeus hispanicus*: SEM micrographs. 40) notogastral cerotegument; a) posterior part of notogaster with mushroom-like cerotegument nodules, b) top view of cerotegument nodules showing irregular border. Scale bar = 10 µm. 41) ventral view. Scale bar =100 µm. 42) microtubercles of cerotegument arranged in rows on border of ventral plate. Scale bar = 2 µm. 43) subcapitulum, ventral view; mentum without foveae, cuticle finely rugose. Scale bar = 20 µm. 44) between rutella (Ru) distal part of lateral lips (Ll), adoral setae as two pairs of conical lappets (arrows); labrum (Lb) in the background. Scale bar = 5 µm.

Remarks / Annotations

Whereas the leg setation is the same in the majority of *Caleremaeus,* few species show some differences (Table 2). Remarkable is the situation in *C. monilipes* of Michaels' slides where both specimens show on tarsi I to III one seta less; the same number of tarsal setae we observed in one specimen of *C. mentobellus*. Interestingly, Norton & Behan-Pelletier [1] reported from one case in *C. arboricolus* where tarsal seta *l"* was also absent on leg I.

Caleremaeus divisus Mihelčič, 1952 represents not a valid species. The figure given in its description, depicting only the anterior half of the dorsal aspect, as well as the short text is not informative. The two diagnostic characters to differentiate it from *C. monilipes* at this time, refer to the distance (without dimensions) between the prodorsal subrectangular sclerotized areas (more or less circular in his figure) and to the shape of the humeral enantiophysis; from the latter Mihelčič [8] wrote that they should be larger than in *C. monilipes* but in his figure they are of the same size as the tubercles of the anterior notogastral border. Also, the ecological information is doubtful. In the species description it was stated that the species was collected from mosses growing on trees but in Mihelčičs' Table 3, referring to oribatids and collembolans of forest soils, *C. divisus* should occur in coniferous litter and "monilipes" in mosses on trees. Neither type material nor other specimens of *C. divisus* exist in the oribatid-mite-collection of Franz Mihelčič in the Tyrolean Museum Ferdinandeum, Innsbruck [9].

Supplementary references – part 2

1. Norton, R. A. & Behan-Pelletier, V. Two unusual new species of *Caleremaeus* (Acari: Oribatida) from eastern North America, with redescription of *C. retractus* and reevaluation of the genus. *Acarologia* **60**(2), 398–448; DOI 10.24349/acarologia/20204375 (2020).

2. Michael, A. D. Further Notes on British Oribatidæ. *J. R. Microscop. Soc.* **2**.1, 1–18 (1882).

3. Grandjean, F. Complément à mon travail de 1953 sur la classification des Oribates. *Acarologia* **7,** 713–734 (1965).

4. Subías, L. S. & Arillo, A. Acari, Oribatei, Gymnonota II. Oppioidea in *Fauna Iberica*, *vol. 15* (eds Ramos A. *et al.*) 289 pp. (Madrid, Museo de Ciencias Naturales, 2001).

5. Ayyildiz, N., Toluk, A., Taskiran, M. & Tasdemir, A. Two new records of the genera *Cepheus* CL Koch, 1835 and *Caleremaeus* Berlese, 1910 (Acari: Oribatida) from Turkey, with notes on their distribution and ecology. *Türk. entomol. bült*. **1**(3), 145–150 (2011).

6. Miko, L. & Travé, J. Hungarobelbidae n. fam., with a description of *Hungarobelba pyrenaica* n. sp. (Acarina, Oribatida). *Acarologia* **37,** 133–155 (1996).

7. Seniczak, A. & Seniszak, St. Morphological ontogeny of *Caleremaeus monilipes* (Acari: Oribatida: Caleremaeidae), with comments on *Caleremaeus* Berlese. *Syst. Appl. Acarol*. **24**, 1995–2009 http://doi.org/10.11158/saa.24.11.3 (2019).

8. Mihelčič, F. Beitrag zur Kenntnis der Oribatei und Collembolen der Humusböden. *Arch. Zool. Ital.* **37**, 93–106 (1952).

9. Totschnig, U. Die Hornmilbensammlung (Acari, Oribatida) Franz Mihelčič im Tiroler Landesmuseum Ferdinandeum, Innsbruck. *Veröff. Tiroler Landesmus. Ferdinandeum* **81,** 205–240 (2001).
